# Costs and its drivers for diabetes mellitus type 2 patients in France and Germany: a systematic review of economic studies

**DOI:** 10.1186/s12913-020-05897-w

**Published:** 2020-11-16

**Authors:** Constance Stegbauer, Camilla Falivena, Ariadna Moreno, Anna Hentschel, Magda Rosenmöller, Tim Heise, Joachim Szecsenyi, Freimut Schliess

**Affiliations:** 1 aQua Institute for Applied Quality Improvement and Research in Health Care GmbH, Maschmühlenweg 8-10, 37073 Göttingen, Lower Saxony Germany; 2 Health & Not for Profit Division, CERGAS, SDA Bocconi School of Management Governments, Via Sarfatti, 10, Milan, 20136 Italy; 3 CRHIM - Center for Research in Healthcare Innovation Management, IESE Business School - University of Navarra, C. d’Arnús i de Garí, 3-7, Barcelona, 08034 Catalonia Spain; 4 Profil, Hellersbergstr. 9, Neuss, 41460 North Rhine-Westphalia Germany

**Keywords:** Cost, Diabetes mellitus type 2, France, Germany, Automated insulin delivery

## Abstract

**Background:**

Type 2 diabetes represents an increasingly critical challenge for health policy worldwide. It absorbs massive resources from both patients and national economies to sustain direct costs of the treatment of type 2 diabetes and its complications and indirect costs related to work loss and wages. More recently, there are innovations based on remote control and personalised programs that promise a more cost-effective diabetes management while reducing diabetes-related complications. In such a context, this work attempts to update cost analysis reviews on type 2 diabetes, focusing on France and Germany, in order to explore most significant cost drivers and cost-saving opportunities through innovations in diabetes care. Although both countries approach care delivery differently, France and Germany represent the primary European markets for diabetes technologies.

**Methods:**

A systematic review of the literature listed in MEDLINE, Embase and EconLit has been carried out. It covered interventional, observational and modelling studies on expenditures for type 2 diabetes management in France or Germany published since 2012. Included articles were analysed for annual direct, associated and indirect costs of type 2 diabetes patients. An appraisal of study quality was performed. Results were summarised narratively.

**Results:**

From 1260 records, the final sample was composed of 24 papers selected according to predefined inclusion/exclusion criteria. Both France and Germany revealed a predominant focus on direct costs. Comparability was limited due to different study populations and cost categories used. Indirect costs were only available in Germany. According to prior literature, reported cost drivers are hospitalisation, prescriptions, higher HbA1c and BMI, treatment with insulin and complications, all indicators of disease severity. The diversity of available data and included costs limits the results and may explain the differences found.

**Conclusions:**

Complication prevention and glycaemic control are widely recognized as the most effective ways to control diabetes treatment costs. The value propositions of self-based supports, such as hybrid closed-loop metabolic systems, already implemented in type 1 diabetes management, are the key points for further debates and policymaking, which should involve the perspectives of caregivers, patients and payers.

**Supplementary Information:**

The online version contains supplementary material available at 10.1186/s12913-020-05897-w.

## Background

The increasing burden of non-communicable chronic diseases represents a critical challenge for healthcare systems. Diabetes is one of the leading public health challenges [1]. The prevalence of the disease, and therefore, diabetes costs are increasing rapidly [2]. In 2014, the global prevalence of diabetes among adults was estimated at 8.5% [3], and it is still growing [4], with the predominance of type 2 diabetes mellitus patients. Due to its impact on everyday activities, diabetes is considered a “prospective disability” [5, 6]. Globally, it represented the direct cause of more than 1.6 million deaths in 2015, and it is expected to become the seventh leading cause by 2030 [7].

Although various therapeutics have demonstrated to be successful at controlling type 2 diabetes, it still represents a mechanistic hub for the evolution of chronic disease cluster and multimorbidity, predisposing to frailty and physical and mental decline. Diabetes and its multidimensional implications involve a large outlay of financial resources for both patients and national economies due to the direct and associated costs of treatment and indirect costs related to loss of work and wages [8]. The steady increase of individuals affected by type 2 diabetes engenders a larger prevalence of related morbidities and a higher risk of mortality, which is the primary concern for healthcare policymakers [9].

Studies aimed at understanding the costs of type 2 diabetes represent valid support to quantify the impact of the disease on society and to support health policy towards value-based diabetes care delivery [10, 11]. Direct and indirect costs of type 2 diabetes have to be considered in order to estimate the societal impact of the disease [12]. Associated costs, mainly composed of the cost of complications, are considered as the primary driver of healthcare expenditure for type 2 diabetes by many scholars [13, 14]. Losses in Gross Domestic Product (GDP) worldwide from 2011 to 2030, including both the direct and indirect costs of diabetes, will be 89 billion Euro in Europe, [8, 10, 15] indicating that the indirect and associated costs are often higher than the direct ones. Therefore, the involvement of all cost categories is critical for performing a worthwhile analysis [16].

Over time, several international studies have dealt with costs estimative of treating diabetes [17–24], pointing out several issues. Whereas in low- and middle-income countries, inadequate access to insulin [25, 26] and oral drugs for controlling glucose and blood pressure represent the main bottleneck in type 2 diabetes treatment; in high-income countries, main problems relate to lifestyle and are correlated with nutrition, exercise habits and obesogenic living environments [27]. Evidence demonstrated that type 2 diabetes is potentially reversible [28, 29] by early diagnosis and higher patient participation in disease management [30]. Accordingly, health policy must deal with the implementation of diabetes management regimens more tailored to individual patient’s treatment-effect-modifiers [31] and aimed at increasing their awareness and satisfaction [32]. Traditional therapies revealed to entail increased costs in the long-term due to the need for treatment intensification after around 8 years and the increased requirement of medication and outpatient visits caused by non-fatal events [9]. Hence, to reduce complications and improve treatment outcomes, there is a demand for a more integrated personalized diabetes management [33].

New drugs combined with digital diabetes technologies that acquire and exploit patient-generated data for individual therapy decisions could critically contribute to the personalization of diabetes management. Recently, an unusually high potential to personalize type 2 diabetes management has been attributed to the use of continuous glucose monitoring tools and automated insulin delivery systems (i.e. artificial pancreas) [34]. These innovative solutions are widely considered fundamental to effectively reduce the risk of hypo- and hyperglycaemic events and the risk of follow-up complications [35]. Furthermore, continuous glucose monitoring contributes to minimize medical errors [36].

However, a successful introduction of such innovations requires careful management of stakeholder expectations and innovation barriers. Several studies have shown that clinical benefits are only achieved when there is a high level of patient adherence over time [37]. Nonadherence and early discontinuation also lead to significant waste of resources [38].

This article is part of an EU-funded project that intends to implement automated insulin delivery systems employing an artificial pancreas for people with type 2 diabetes in France and Germany [34]. Carrying out a systematic literature review, it attempts to identify the most relevant cost drivers in type 2 diabetes management in France and Germany. Both countries represent together the largest markets for diabetes technologies in Europe and offer the best contexts for testing new solutions. In 2015 in Germany alone, there were 6.9 million people (corresponding to 7–8% of the adult population) with diagnosed diabetes. The estimative suggested that another 2 million people had undiagnosed diabetes. By 2040, additional 3.8–5.4 million people are projected to have diabetes. There is a huge regional heterogeneity, with 11.6% prevalence in East- and 8.9% in West-Germany [39]. Also, data from France reveal an increasing prevalence of type 2 diabetes [40]. It has been estimated that there are more than 3.4 million cases of diabetes in adults (representing 7.6% of the adult population) similarly distributed in all French regions, with a significant presence of people aged ≥45 years (94% over the total cases). Fuentes et al. explored the evolution of type 2 diabetes in France between 2010 and 2017. They appraised that the crude prevalence of diabetes has increased from 10.9 to 11.8% for men and from 7.9 to 8.4% for women [41]. Furthermore, there are still numerous cases of undiagnosed diabetes due to the scanty adoption of screening programmes [40]. Several advanced devices that involve an automatic insulin infusion and self-management systems are already available on under evaluation in France [42]. Also, in Germany digital solutions including continuous glucose monitoring, smart insulin pens as well as automated insulin delivery systems play an increasing role in diabetes self-management [43]. As recognized all over the world, such improvements in managing diabetes and prevent asymptomatic adverse events have been contributing at reducing mortality rates [44, 45].

However, some differences in terms of costs arise between France and Germany. Indeed, although both are Bismarckian systems, the two countries approach healthcare provision differently, which makes it possible to address the different needs depending on the healthcare system characteristics. While the German health system is based on a “fee for service” reimbursement system, the French one is closer to the Beveridgian system, where services are reimbursed using the diagnosis-related group method (DRG).

In addition to updating the cost analysis for type 2 diabetes and the most significant cost drivers, this work aims at paving the theoretical basis for defining a value proposition for innovative solutions, such as artificial pancreas systems in the treatment of people with type 2 diabetes. Recognizing all the costs represents an earlier step towards providing higher benefits to payers and patients.

## Methodology

### Systematic search

A systematic review of the literature was carried out to identify German and French type 2 diabetes costs.

We searched for studies meeting the following predefined inclusion/exclusion criteria:

- Language: to allow replicating the process and avoid missing relevant studies in the specific contexts, studies published in English, French or German were included.
- Time frame: because of the last cost analysis of type 2 diabetes was published in 2012, our analysis refers to papers published after 01/01/2012.
- Topic: to be consistent with the aims explained above, the selection of articles focused on reporting direct or indirect type 2 diabetes costs for France or Germany.
- Source: journal articles; case studies, case series, notes, conference abstracts, editorials, letters, methodological studies and any grey literature were removed to ensure a high quality of the selection process.
- Type of publication: original articles with abstract were included for the first-level screening on the abstract content.

The systematic literature search was conducted in MEDLINE, Embase (both via Embase) and EconLit (via EBSCOhost). A search strategy was developed for each search interface. We used available controlled vocabulary, keywords/headings and limits. The search strategies were based on a combination of terms for type 2 diabetes, for expenditures and for France or Germany, respectively. The terms were combined with Boolean Operators AND or OR. If possible, the inclusion/exclusion criteria were used (for detailed search strategies, see Tables 1 and 2; Additional material). The searches were conducted on June 5th, 2019.

### Selection process

Applying the aforementioned criteria each hit was screened for eligibility by two independent researchers, first only by title and abstract. In a second step, if they seem to fit or if a decision was not possible based on the abstract, they were also screened by full text. Four researchers participated in the screening process. Discrepancies were discussed and resolved jointly.

### Data collection and analysis

For all included papers, the following data was extracted:

- author(s), title and publication year
- country
- study design
- description of population
- number of included patients
- source of cost data
- year(s) of reported costs
- performed statistical analyses
- different kind of reported costs, cost classes (overall direct healthcare, diabetes-specific direct, diabetes-associated direct and indirect costs)
- all reported annual costs per patient

One researcher gathered the data in an excel spreadsheet that was used for the analysis and narrative summary of the costs. All costs were converted and adjusted to December 2018 Euro (€) using Statbureau [46]. We used July of the reported costs year as a starting month for this adjustment or the median month if there was a longer or shorter time period. If there were studies that provide no costs that could be extracted as annual cost per patient, the study was excluded post hoc.

The analysis and summary of the data were planned as narrative synthesis. The approach of this systematic review does not allow a meta-analysis because no specific intervention is in the focus of this review and included studies would not report any effect sizes. Instead, we aimed at summarising all findings on healthcare costs for France and Germany for type 2 diabetes patients. This broad approach led us to not restrict our search strategy to specific study designs, being open to any kind of type 2 diabetes patient population and including all studies reporting any kind of healthcare costs related to patients with type 2 diabetes. We expected great data heterogeneity, which makes any statistical analysis that include all studies difficult. Therefore, we decided to conduct a narrative synthesis of the data and support this synthesis with tables aggregating data, where possible [47].

### Cost classification

The analysis differentiates the direct and indirect costs of the disease. Direct costs are those generated by the condition itself (e.g. hospital admissions, drugs, specialist and general practitioners’ visits, services for measuring blood glucose level and administering insulin, transport, rehabilitation). To better evaluate the economic magnitude of type 2 diabetes, direct costs are considered as:

- overall direct healthcare costs when they include all direct costs of any consumption of healthcare services, also including costs that are not related to diabetes (e.g. vaccination, treating a broken arm);
- diabetes-specific direct costs for diabetes treatment when they directly affect the consumption of inpatient and outpatient care for diabetes treatment, e.g. diabetes-related physician visits, as well as antihyperglycemic medication and devices and material for blood glucose monitoring;
- diabetes-associated direct costs for additional services related to consequences of type 2 diabetes, usually specialist visits to monitor correlated problems and health services delivered to prevent complications, i.e. diseases and symptoms that might emerge from diabetes, for example renal diseases, diabetic foot syndrome and amputations.

Indirect costs are represented by the share of present and future loss of productivity due to the disease, such as reduced income from work, lost working days, disability, early retirement and premature death [17, 48]. The cost classification was carried out based on the information on included data as well as data sources presented in the method section of the included papers.

### Quality assessment

Since there is no generally accepted method to assess the quality of economic studies [49], we decided to follow the recent proposal from the British Medical Journal Checklist for economic submissions [50]. This quality assessment consists of the following 10 criteria related to specific aspects of cost reporting articles:

1. Was a clear definition of the illness given?
2. Were epidemiological sources carefully described?
3. Were direct/indirect costs sufficiently disaggregated?
4. Were activity data sources carefully described?
5. Were activity data appropriately assessed?
6. Were the sources of all cost values analytically described?
7. Were unit costs appropriately valued?
8. Were the methods adopted carefully explained?
9. Were the major assumptions tested in a sensitivity analysis?
10. Was the presentation of results consistent with the methodology of the study?

We extracted information from these 10 criteria and examined if the criteria were fully, not, or partially met. Based on this assessment, a score was calculated for each criterion (fully met = 1 point, partially met = 0.5 points, not met = 0 points), and the scores aggregated for each paper. The range of this score is 0–10, with higher scores indicating better quality. Results of quality assessment are reported in the results section but were not used to exclude studies.

## Results

### Search results

**Fig. 1:**
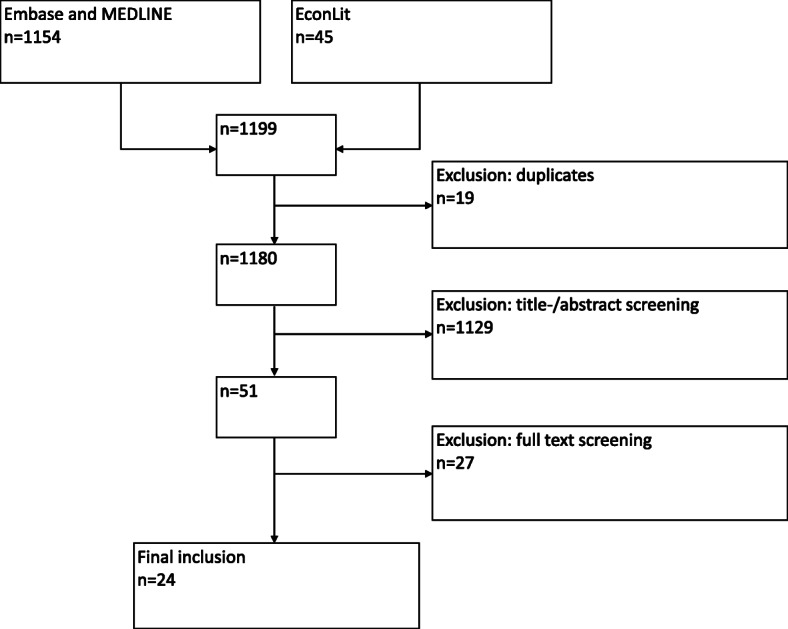
Selection process of the systematic literature search

Our search strategy resulted in 1260 hits, including 19 duplicates. Hence, 1241 papers were included in the selection process, leaving 51 articles for full text screening. The selection process resulted in 24 papers for inclusion. Figure 1 documents this process.

### Included papers

Of these 24 papers, nine papers report 165 cost items on diabetes costs in France [51–59] (all direct costs) and 16 papers report 135 cost items on diabetes costs in Germany of which 131 describe direct costs [36, 59–73]. Cost assessment was based on different numbers of patients ranging from 32 patients in a study performing a survey [72] up to 2.7 million patients in a study analysing health insurance claims data [62]. The population varied regarding age and treatment. Furthermore, one study only included employed patients [63]. Five studies did not assess the actual costs but used models to estimate costs [36, 61, 70–72]. These studies often refer to previous studies for incidence rates and costs. Most studies based their cost assessment on secondary data, e.g. statutory health insurance data. Two studies collected data prospectively [65, 74]. The year of reported costs varied between 2005 and 2017 (for detailed information on the included studies, see Table 3; Additional material).

### Results of quality assessment

The included studies have scores between four [62] to nine points [51, 65, 69, 70]. No paper hit all 10 criteria. Fifteen studies reached eight or more points. Most studies presented their results as described in the methodology part and appropriately valued unit costs. Five studies performed a sensitivity analysis of their major assumption (for detailed quality assessment results, see Table 4; Additional material).

### Diabetes costs in Germany

All German studies included direct costs [36, 59–73], one study also assessed indirect costs [64] (for detailed results, see Tables 5–20; Additional material). Except for indirect costs, all costs were presented based on the perspective of statutory health insurances, including only costs that are reimbursed by them.

**Fig. 2:**
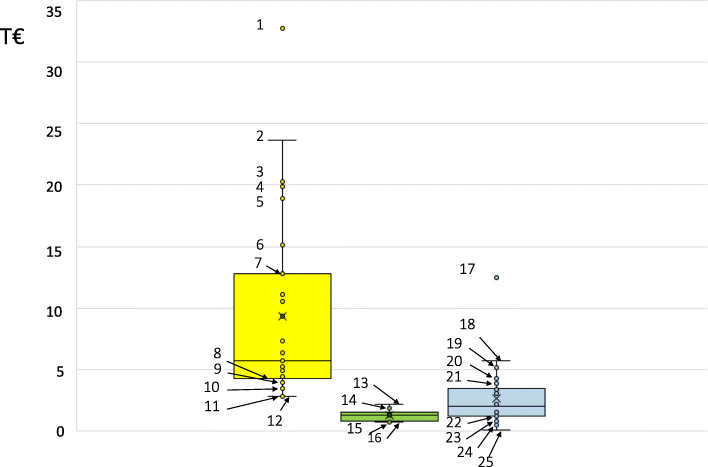
Box and Whisker plot representations of direct type 2 diabetes costs in Germany. Yellow plot, overall direct healthcare costs; green plot, diabetes-specific direct costs; blue plot, diabetes-associated direct costs. T€, thousands of Euro per patient and year, as reported in [36, 60–66, 69, 70, 72, 73] and indicated in Tables 5–8, 10–14, 16–18 and 20, Additional materials. Outliers and values larger or equal to the upper limit of the third / less or equal to the lower limit of the first quartile. 1, end-stage renal disease (first year) [61]; 2, end-stage renal disease (second year) [61]; 3, last year of life for patients in a type 2 diabetes disease management program [60]; 4, fatal ischemic heart disease [61]; 5, last year of life for patients not in a type 2 diabetes disease management program [60]; 6, nonfatal stroke [61]; 7, fatal stroke [61]; 8, foot complications [61]; 9, enrolled in a type 2 diabetes disease management program [60]; 10, overall healthcare direct costs [64]; 11, retinopathy [61]; 12, no complication [61]; 13, 1 year after insulin initiation [65]; 14, 6 months prior and after insulin initiation [65]; 15, prior insulin glargine initiation [66]; 16, prior NPH insulin initiation [66]; 17, myocardial infarction [36]; 18, on insulin only, excess costs [64]; 19, myocardial infarctions (follow-up, first year) [36]; 20, at least one urinary tract infection, excess costs [73]; 21, disease duration ≥20 years, excess costs [64]; 22, 6.5 ≤ HbA1c < 7.5%, excess costs [64]; 23, disease duration 3–10 years, excess costs [64]; 24, without pharmacological treatment, excess costs [64]; 25, severe hypoglycaemic events (~ 0,1 severe hypoglycaemic events per patient per year) [72]

Direct costs – overall healthcare costs: Five studies did not limit their cost analysis to costs of type 2 diabetes treatment but included all expenses for any healthcare of type 2 diabetes patients [60–64]. These five studies reported overall direct healthcare costs for 33 different patient population, with most of these ranging between €2793.33 for type 2 diabetes patients without any complication [61] and up to €4882.11 overall direct healthcare costs for an average type 2 diabetes patient [62] (Table 1, Fig. 2). Few outliers for high costs were found (Table 1, Fig. 2) with up to 6-fold of costs for an average type 2 diabetes patient: patients with type 2 diabetes and end-stage renal disease [61], patients with type 2 diabetes and an amputation [61] and patients with type 2 diabetes in their last year of life [60]. Two studies reported overall direct healthcare costs for type 2 diabetes patients for different services and resources: inpatient care, outpatient care and medication (for details, see Table 1), with higher costs for inpatient care and medication than for outpatient care [63, 64].

**Table 1:** Summary of costs in Germany from the included studies

	Range of reported costs	References
**Overall direct healthcare costs**		
Total	€2793.33 – €4882.11	[60–64]
End-stage renal disease	€32,738.14 – €23,629.17	[61]
Amputation	€20,512.96 – €12,818.02	[61]
Last year of life	€18,874.05 – €20,249.61	[60]
Inpatient care	€1142.20 – €1728.90	[63, 64]
Outpatient care	€496.65^a^ – €766.17	[63, 64]
Medication	€997.44 – €1172.75	[63, 64]
**Diabetes-specific direct costs**		
Total	€774.66 – €2204.41	[59, 65, 66]
Inpatient care	€83.82 – €176.02	[59, 65]
Outpatient care	€438.25^a^ – €562.78^a^	[59, 65]
Antihyperglycemic treatments	€288.20 – €1887.27	[59, 65, 67, 68]
Blood glucose measurement	€638.21 – €943.55	[59, 65]
**Diabetes-associated direct costs**		
Total	€499.49 – €5724.91	[64, 69–71]
Hypoglycaemic episode	€98.91 – €2966.70^a^	[36, 72]
Myocardial infarction	€5138.78 – €12,448.04	[36]
Urinary tract infection	€4253.26	[73]
Inpatient care	€776.13	[64]
Outpatient care	€151.70^a^	[64]
Medication	€500.80	[64]
**Indirect costs**		
total	€4263.02	[64]
Inability to work	€3474.42	[64]
Indirect excess costs	€2204.76	[64]
Inability to work – excess costs	€2124.76	[64]

^a^These costs are the sum of different unit costs and are not listed in additional material

Direct costs – diabetes-specific costs: Another five studies reported diabetes-specific costs [59, 65–68]. Total direct diabetes-specific costs ranged between €774.66 in the year prior to insulin initiation use for patients discontinuing this treatment within 3 months [66] and €2204.41 for patients in the year after insulin initiation [65]. No extreme outlier was found for this cost category (Fig. 2). Table 1 also shows the cost range found in the included studies for inpatient care, outpatient care, antihyperglycemic treatments and blood glucose measurement, with highest costs for antihyperglycemic treatments and blood glucose measurement.

Direct costs – diabetes-associated costs: Seven studies reported diabetes-associated costs [36, 64, 69–73]. Reported diabetes-associated costs were excess costs comparing direct healthcare costs for patients with and without type 2 diabetes or costs for diabetes-associated complications. Annual excess costs for type 2 diabetes were estimated to be €499.49 for patients with type 2 diabetes and no antihyperglycemic treatment and range up to €5724.91 for insulin treated patients with type 2 diabetes [64] (Table 1, Fig. 2). The comparison of annual healthcare costs of type 2 diabetes patients with and without urinary tract infection results in €4253.26 overall healthcare excess costs [73] (Table 1, Fig. 2). Reported costs for myocardial infarction of type 2 diabetes patients ranged between €5138.78 and €12,448.04 [36] and reported costs for hypoglycaemic episodes between €98.91 and €2966.70 [36, 72] (Table 1, Fig. 2). Diabetes-associated direct costs for inpatient care, outpatient care and medication were reported as excess costs in one study [64] (for details, see Table 1).

Indirect costs: Indirect costs were assessed by one study [64]. Excess costs for patients with and without type 2 diabetes were estimated based on a survey of the working population aged ≤65 years. Annual indirect costs (sick leaves, incapacity benefits) for this type 2 diabetes population were €4263.02, including €3474.42 for sick leaves. With this, type 2 diabetes people caused €2204.76 indirect excess costs, including €2124.76 for sick leaves.

### Diabetes costs in France

Nine studies report costs for type 2 diabetes patients in France [51–59]. They report annual overall healthcare costs, as well as specific costs for diabetes treatment and associated costs (for detailed results, see Tables 21–29; Additional material). No study assessed indirect costs. Seven studies based their analysis on the same database: L’Échantillon Généraliste de Bénéficiaires [52–58].

**Fig. 3:**
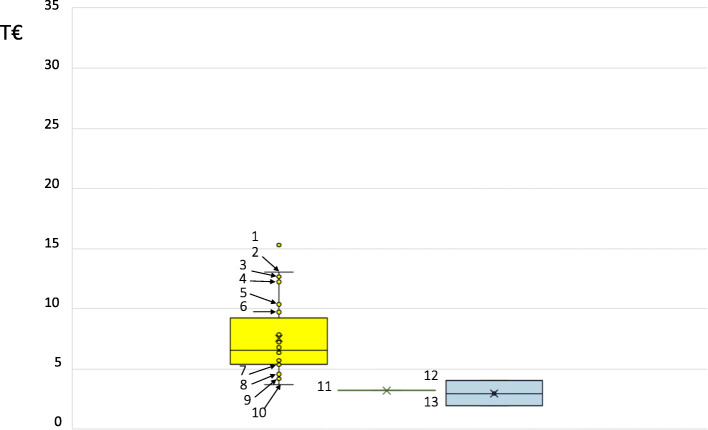
Box and Whisker plot representations of direct type 2 diabetes costs in France. Legend: Yellow plot, overall direct healthcare costs; green plot, diabetes-specific direct costs; blue plot, diabetes-associated direct costs. T€, thousands of Euro per patient and year, as reported in [51–57, 59] and indicated in the Additional materials (Tables 21–24 and 26–29). Outliers and values larger or equal to the upper limit of the third / less or equal to the lower limit of the first quartile. 1, year after insulin initiation, < 60 years of age [55]; 2, overall healthcare direct costs [53]; 3, 1 year after insulin initiation, 60–75 years of age [55]; 4, Ton insulin [54]; 5, year before insulin initiation, < 60 years of age [55]; 6, year after insulin initiation, ≥75 years of age [55]; 7, 3 years before insulin initiation, 60–75 years of age [55]; 8, 2 years before insulin initiation, ≥75 years of age [55]; 9, on metformin plus DPP4 inhibitor [56]; 10, on metformin plus sulfonylurea [56]; 11, diabetes-specific direct costs: diabetes treatment (total) [59]; 12, overall healthcare direct costs, excess costs [53]; 13, overall healthcare direct costs, excess costs [51]

Direct costs – overall healthcare costs: Seven studies report healthcare costs for type 2 diabetes patients [51–57] for 23 different patient population ranging between €3717.22 for patients treated with metformin and sulfonylureas [56] and €15,299.46 in the year after insulin initiation for patients younger than 60 years of age [55] (Table 2, Fig. 3). Five or six studies respectively, reported overall direct healthcare costs for inpatient care, outpatient care and medication, with highest costs of outpatient care (for details, see Table 2). Additional costs were reported by five studies [51–55], including for example costs of transportation or laboratory tests (for details, see Tables 21–29; Additional material).

**Table 2:** Summary of costs in France from the included studies

	Range of reported costs	References
**Overall direct healthcare costs**		
Total	€3717.22 – €15,299.46	[51–57]
Inpatient care	€940.49 – €4542.94	[51–56]
Outpatient care	€2303.53 – €8749.88	[51, 52, 54–56]
Medication	€162.84 – €2798.49	[51–56]
**Diabetes-specific direct costs**		
Total	€3229.75	[59]
Inpatient care	€1366.39 – €4926.54	[58, 59]
Outpatient care	€819.60^a^	[59]
Antihyperglycemic treatments	€727.08^a^	[59]
Blood glucose measurement	€315.50	[59]
**Diabetes-associated direct costs**		
Total	€1958.33 – €4050.45	[51–53]
Inpatient care	€549.45 – €5333.19	[51–53, 58]
Outpatient care	€1306.24 – €2070.65	[51, 52]
Medication	€808.21 – €845.80	[52, 53]
**Indirect costs**	No information available	

^a^These costs are the sum of different unit costs and are not listed in additional material

Direct costs – diabetes-specific costs: One study [59] reported diabetes-specific direct costs. €3229.75 total costs were reported for 6 months before and after insulin initiation, including €1366.39 for diabetes-related hospitalisations, €709.29 for physician consultations, €476.81 for oral antidiabetics and €250.27 for insulin as well as €315.50 for blood glucose monitoring. Another study reported costs for inpatient care with a diagnosis of hypoglycaemia as the main diagnosis with up to €4926.54 annual inpatient costs [58].

Direct costs – diabetes-associated costs: Associated healthcare costs were assessed as excess costs by three studies [51–53], ranging between €1958.33 for patients newly treated for type 2 diabetes [51] and €4050.45 for patients treated with insulin [53] (Table 2, Fig. 3). Diabetes-associated costs of inpatient care, outpatient care and medication were also reported in more detail with higher costs of outpatient care (for details, see Table 2). Additionally, one study reported costs of up to €5333.19 of any hospitalisation with a hypoglycaemia [58]. Indirect costs: No included study reported indirect costs for France.

### Cost drivers in France and Germany

Looking at healthcare costs for type 2 diabetes patients, highest healthcare expenditures were caused by hospitalisation and prescriptions [52–54, 56, 62, 64]. When only costs directly attributable to type 2 diabetes treatment were considered, hospitalisation (here diabetes-related hospitalisation) is no longer the main cost driver, especially in patients with insulin therapy in Germany [65]. Instead, costs for blood glucose self-management and prescriptions contribute the most to type 2 diabetes treatment costs [59]. Whereas in France diabetes-related hospitalisations followed by prescriptions remain the main cost driver [59].

In Germany, a trend of higher antihyperglycemic treatment costs can be seen in patients with higher HbA1c (€288.20 for HbA1c < 6.5; €882.23 for HbA1c ≥9) and higher BMI (€405.35 for BMI < 30; €718.43 for BMI ≥35) [67]. Higher overall direct healthcare costs were reported for patients with longer disease duration [64]. In both France and Germany, costs for patients using insulin were higher than for patients not on insulin [54, 55, 59, 63–66].

Complications or comorbidities contribute to higher overall direct healthcare costs and direct costs of diabetes treatment, respectively [61, 67]. In Germany, the most expensive diabetes-related complications are end-stage renal disease (occurrence €32,738.14, one-year follow-up €23,629.17), amputation (occurrence €20,512.96, one-year follow-up €12,818.02) and fatal ischaemic heart disease (occurrence €19,874.15) [61]. No comparable studies were found on France.

A few studies also analysed direct costs for different sex and age groups, but no explicit trends became apparent. Three German studies stratified direct costs for different age groups with the result of higher costs for younger patients (€568.11 for patients aged ≤60 years vs. €402.24 for patients aged > 80 years) [67] and for older patients [63, 69]. One French study also reported higher costs for younger patients (€15,299.46 for patients aged < 60 years vs. €9728.25 for patients aged ≥75 years) [55]. Similar heterogeneous results were found for sex: higher costs for men [67] and also higher costs for women [63].

## Discussion

The objective of this study was to assess and compare the costs of type 2 diabetes in France and Germany and emphasise cost drivers in order to understand which issues must be addressed by innovators in diabetes care to improve patient care and coincidently reduces costs. The decision to focus on France and Germany, therefore, was made because these countries represent significant share of health technology innovation markets in Europe and entail various needs due to differences in healthcare provision.

Among the 24 studies included, a higher number of studies were investigating a German cohort than a French one (respectively, 16 and 9). The higher number of people with diabetes and the higher diversity of reimbursement mechanisms [75] may explain the higher interest around the type 2 diabetes costs in Germany. The analysis was carried out evaluating direct and indirect costs related to type 2 diabetes. While direct costs refer to resources specifically employed in inpatient and outpatient treatment, indirect costs measure the share of present and future loss of productivity due to the disease. Diabetes-associated costs, mainly related to complications, are often estimated as excess costs, which provide valuable information on the contribution of type 2 diabetes to the overall disease burden on healthcare expenditure, by several authors [51–53, 64, 69, 71, 73].

The results of this literature review show large differences in reported costs, for example between average annual overall direct healthcare costs (€2793-€32,738 in Germany and €3717–€15,299 in France). Whereas excess costs seem quite similar (€499–€5724 in Germany vs. €1958–€4051 in France). Only one included study [64] assessed indirect costs of type 2 diabetes.

The scarce attention to indirect costs reveals an important gap in the health policy debate as the treatment of diabetes represents a critical issue for the overall sustainability of healthcare systems. Moreover, it underlines a lack of studies aimed to explore patients’ standpoint in order to better identify room for innovation in type 2 diabetes that would go beyond glycaemic management and improve the quality of life of people with type 2 diabetes.

Since the search included any study regarding the costs of type 2 diabetes patients, heterogeneous studies in terms of the patient population, data sources and cost categories were included. These different approaches in the design of the studies allow a comprehensive picture of costs for type 2 diabetes but concurrently limited the immediate comparison of costs and seem to be one major reason for costs differences. The different approaches in healthcare delivery of the two national health systems also contribute to these differences. Germany has an universal single-payer system funded by statutory health insurances and private insurances. All citizens and permanent residents must subscribe to health insurance. Hence, data included into studies are inferred from statutory health insurance databases that allows to better identify the various cost items. France, instead, has a universal healthcare system mostly paid by government national health insurance, which covers 70–75% of health expenditure. Most of the included studies from France and Germany are based on data claims from these insurances, based on their perspective.

The update of cost analysis on type 2 diabetes, on the one hand, has underlined once again that disease progression towards the presence of complications is the main cost driver. Results on age and sex are not that clear-cut. On the other, it has indicated the potential areas for cost savings, which may be addressed by enforcing preventative care and the implementation of more advanced technologies. High costs were caused by hospitalisations and prescriptions, which are firmly correlated to diabetes complications and comorbidities [14]. As asserted by several authors [13, 76], the prevention of complications represents the primary policy measure to decrease expenditure for diabetes. Besides, the cost of hospitalisation may be decreased by a gradual shift from the hospital setting to ambulatory care in the management of diabetes [77]. However, past experiences do not provide robust evidence on whether ambulatory care could reduce type 2 diabetes costs. On the one hand, ambulatory care delays the progression of chronic diseases, reducing the risk of costly episodes of inpatient care; on the other, it might prompt additional investigations, increasing the probability of hospitalisation [78].

A future-oriented perspective identifies the self-monitoring of blood glucose as a key pillar of type 2 diabetes management [35, 79]. Such an approach underlines the need for integrated personalized diabetes management [33]. This would require interactive collaboration between users, healthcare providers, and payers and included structured training and education for type 2 diabetes patients. To that end, telemedicine can facilitate integrated management of diabetes by enhancing communication between patients and healthcare providers and the continuous sharing of real-life data [36]. Automated insulin delivery (artificial pancreas systems) and also digitally enhanced technologies for multiple-daily insulin administration, such as smart pens in combination with continuous glucose monitoring, are arising as cost-effective solutions in type 2 diabetes management [80]. Over the time, similar technologies grounded on telehealth indeed revealed to be an essential tool for filling the lack of personalized support in daily life [31], producing significant improvement in HbA1c levels and in reducing diabetes-related complications than usual care [81].

The development of such innovative systems is heavily influenced by the needs perceived by patients, but its implementation also depends on the availability of reimbursement [13]. Accordingly, probing direct and indirect costs of treating type 2 diabetes represents a former attempt of designing a reimbursement strategy. The purpose is to develop a tariff that covers actual disbursement of resources and enhances the benefit for patients.

From a value-based healthcare point of view innovations in diabetes care should improve patient-reported outcomes while making type 2 diabetes management more affordable for healthcare systems by tackling the identified cost drivers. In fact, innovative solutions should delay disease progression. E.g. automated insulin delivery systems by means of an artificial pancreas could help to keep personalized glycaemic control targets thereby minimising complications and the need for polypharmaceutical treatment. This should help to reduce costs resulting from healthcare utilisation for complication and from medication consumption and blood glucose monitoring.

Several limitations undermine the usefulness of the results achieved here. First, the scarce generalization of findings that are strongly influenced by specific national health systems and reimbursement policies. Besides, the wide range of methods and patients’ populations involved in the studies, that did not allow to adopt a common framework for the cost analysis. Lastly, while some studies analysed the total costs, other studies focused on single parts of the care or specific patients’ groups. Nevertheless, the different approaches in providing healthcare underline the importance to involve both healthcare provider characteristics [82] and patients perspective [83] in developing an effective value proposition to tackle type 2 diabetes management.

## Conclusions

The study pointed out that most significant cost drivers are represented by hospitalization and complications, which may be stemmed by the employment of remote control technologies and innovative services. In addition to reducing diabetes-related expenditure, these novel solutions can benefit patient’s daily life, enhancing their autonomy. Efforts by policymakers should address promotion of these patient-centred treatments by developing ad hoc policies and reimbursement tariff.

## Supplementary Information

**Additional file 1.** The additional material includes the documentation of the systematic searches (Tables 1-2), detailed information on the included papers (Table 3), the results of the quality assessment of all included papers (Table 4) as well as an overview of the results for each included paper (Tables 5–29).

## Data Availability

Data extracted from the included papers and used for the analysis are provided as additional materials of this manuscript.

## Abbreviations

BMI: Body Mass Index
DPP: dipeptidylpeptidase
GDP: Gross Domestic Product
HbA1c: Glycated haemoglobin
NPH: Neutral Protamine Hagedorn
