# Supplementary material for: Costs and its drivers for diabetes mellitus type 2 patients in France and Germany: a systematic review of economic studies
Source: BMC Health Serv Res. 2020 Nov 16;20:1043. doi: 10.1186/s12913-020-05897-w (PMC7667793; doi:10.1186/s12913-020-05897-w)
Supplement: Supplementary file 1 — Additional file 1. The additional material includes the documentation of the systematic searches (Tables 1-2), detailed information on the included papers (Table 3), the results of the quality assessment of all included papers (Table 4) as well as an overview of the results for each included paper (Tables 5–29). [file 12913_2020_5897_MOESM1_ESM.docx]

**Additional Material:**

Table 1: Documentation of the search strategy in Embase (incl. Medline) from June 5^th^, 2019

| **Number** | **Query** | **Results** |
| --- | --- | --- |
| #1 | france AND [1-1-2012]/sd | 626,953 |
| #2 | french AND [1-1-2012]/sd | 174,676 |
| #3 | germany AND [1-1-2012]/sd | 1,169,004 |
| #4 | german AND [1-1-2012]/sd | 234,968 |
| #5 | europe AND [1-1-2012]/sd | 107,087 |
| #6 | european AND [1-1-2012]/sd | 978,483 |
| #7 | #1 OR #2 OR #3 OR #4 OR #5 OR #6 | 2,478,825 |
| #8 | diabetes:ti,ab,kw AND [1-1-2012]/sd | 394,032 |
| #9 | T2dm:ti,ab,kw AND [1-1-2012]/sd | 25,900 |
| #10 | ‘type 2 dm’:ti,ab,kw AND [1-1-2012]/sd | 3,481 |
| #11 | T2d:ti,ab,kw AND [1-1-2012]/sd | 13,525 |
| #12 | Dm2:ti,ab,kw AND [1-1-2012]/sd | 2,318 |
| #13 | Niddm:ti,ab,kw AND [1-1-2012]/sd | 448 |
| #14 | ‚metabolic disorder‘:ti,ab,kw AND [1-1-2012]/sd | 4,710 |
| #15 | ‚metabolic disease‘:ti,ab,kw AND [1-1-2012]/sd | 6,958 |
| #16 | #8 OR #9 OR #10 OR #11 OR #12 OR #13 OR #14 OR #15 | 404,285 |
| #17 | Expenditure:ti,ab,kw AND [1-1-2012]/sd | 26,505 |
| #18 | ‘burden of illness’:ti,ab,kw AND [1-1-2012]/sd | 1,523 |
| #19 | ‘burden of disease’:ti,ab,kw AND [1-1-2012]/sd | 8,513 |
| #20 | Boi:ti,ab,kw AND [1-1-2012]/sd | 125 |
| #21 | coi:ti,ab,kw AND [1-1-2012]/sd | 4,174 |
| #22 | cost:ti,ab,kw AND [1-1-2012]/sd | 274,324 |
| #23 | #17 OR #18 OR #19 OR #20 OR #21 OR #22 | 307,623 |
| #24 | #7 AND #16 AND #23 | 3,295 |
| #25 | #24 NOT [conference abstract]/lim | 1,308 |
| #26 | #25 NOT [animals]/lim | 1,162 |
| #27 | #26 AND [abstracts]/lim | **1,154** |

Table 2: Documentation of the search strategy in Econlit from June 5th, 2019

| **number** | **query** | **results** |
| --- | --- | --- |
| #1 | france Limiters – Published Date:20120101 | 8,870 |
| #2 | french Limiters – Published Date:20120101 | 4,414 |
| #3 | germany Limiters – Published Date:20120101 | 13,194 |
| #4 | german Limiters – Published Date:20120101 | 7,239 |
| #5 | europe Limiters – Published Date:20120101 | 99,100 |
| #6 | european Limiters – Published Date:20120101 | 38,217 |
| #7 | S1 OR S2 OR S3 OR S4 OR S5 OR S3 | 120,401 |
| #8 | TI diabetes OR AB diabetes OR SU diabetes Limiters – Published Date:20120101 | 267 |
| #9 | TI t2dm OR AB t2dm OR SU t2dm Limiters – Published Date:20120101 | 4 |
| #10 | TI ‘type 2 dm’ OR AB ‘type 2 dm’ OR SU ‘type 2 dm’ Limiters – Published Date:20120101 | 5 |
| #11 | TI t2d OR AB t2d OR SU t2d Limiters – Published Date:20120101 | 3 |
| #12 | TI dm2 OR AB dm2 OR SU dm2 Limiters – Published Date:20120101 | 2 |
| #13 | TI niddm OR AB niddm OR SU niddm Limiters – Published Date:20120101 | 0 |
| #14 | TI ‚metabolic disorder‘ OR AB ‚metabolic disorder‘ OR SU ‚metabolic disorder‘ Limiters – Published Date:20120101 | 2 |
| #15 | TI ‚metabolic disease‘ OR AB ‚metabolic disease‘ OR SU ‚metabolic disease‘ Limiters – Published Date:20120101 | 3 |
| #16 | S8 OR S9 OR S10 OR S11 OR S12 OR S13 OR S14 OR S15 | 274 |
| #17 | TI ‚burden of illness‘ OR AB ‚burden of illness‘ OR SU ‚burden of illness‘ Limiters – Published Date:20120101 | 20 |
| #18 | TI ‚burden of disease‘ OR AB ‚burden of disease‘ OR SU ‚burden of disease‘ Limiters – Published Date:20120101 | 155 |
| #19 | TI expenditure OR AB expenditure OR SU expenditure Limiters – Published Date:20120101 | 25,014 |
| #20 | TI cost OR AB cost OR SU cost Limiters – Published Date:20120101 | 61,389 |
| #21 | TI boi OR AB boi OR SU boi Limiters – Published Date:20120101 | 11 |
| #22 | TI coi OR AB coi OR SU coi Limiters – Published Date:20120101 | 19 |
| #23 | S17 OR S18 OR S19 OR S20 OR S21 OR S22 | 82,268 |
| #24 | S7 AND S16 AND S23 | **45** |

Table 3: Detailed information on the included papers

| **Reference** | **Country** | **Information on patient population** | | **Information on costs** | | | | |
| --- | --- | --- | --- | --- | --- | --- | --- | --- |
|  |  | **Description of patient population** | **Number of included patients** | **Type of costs*** | **Included costs** | **Data sources** | **Year(s) of reported costs** | **Reported costs (annual costs per patient)** |
| Anderten et al. 2015 | Germany | T2D, initiation of insulin glargine or neutral protamine hagedorn insulin | 2,765 patients with glargine insulin, 1,554 patients with neutral protamine hagedorn insulin | direct | antidiabetic drugs, consumables (e.g. test strips), medication for hypoglycaemia, diabetes-related: physician visits, therapeutic remedies and aids, diabetes education, diagnostic procedures | Disease Analyser database (IMS HEALTH),  Lauer Taxe/ EBM | 01/2008 – 03/2014 | For each type of insulin as well as prior and post insulin initiation as well as for persistent and discontinuation patients:  total costs,  diabetes related prescription costs,  antidiabetic medication costs,  consumables costs,  costs for treatment of hypoglycaemia,  costs for other medical services |
| Baudot et al. 2019 | France | T2D, ≥ 45 years of age and antidiabetic medication, incident patients in 2008 | 170,013 patients with T2D, 510,022 patients without T2D | Direct, associated | primary care (outpatient care, nursing care, drugs, medical devices and services, transportation physiotherapy), cash benefits (sickness and disability benefits), hospital care (public and private hospitals) | National health insurance administrative database | 2015 | Average annual per capita costs for: general practitioner care, other specialist care, dental care, midwifery, physiotherapy, nursing care, other paramedical care, clinical pathology, drugs, medical devices and services, transportation, other primary care, public hospital care, private hospital care, sickness benefits and work accidents/ occupational diseases, disability benefits |
| Charbonnel et al. 2018 | France | T2D patients with antidiabetic medication | 25,987 patients with T2D, 76,406 patients without T2D | Direct, associated | Medication, reimbursed pharmacy products, hospitalisation, consultations, paramedical care, laboratory tests, medical devices, transport | L’Échantillon Généraliste de Bénéficiaires  (General Sample of Beneficiaries) | 2013 | Average annual per capita costs for: hospital costs, medication, physician consultation, home visits, Interventions, nursing care, Physiotherapy, medical devices, dental care, laboratory tests, transportation, overall costs, ambulatory costs, excess costs |
| Detournay et al. 2015 | France | T2D patients treated with insulin over one year (excluding incident cases, cases that died in that year, cases released during that year from the base, patients reporting to locals mutualist) | n=3,787 | direct, associated | Costs for hospitalisation, other services, transport, medical goods and related services (la liste des produits et prestations), biology, dental fees, therapeutic remedies and aids, drugs, medical fees | L’Échantillon Généraliste de Bénéficiaires  (General Sample of Beneficiaries) | 2013 | Costs for: hospitalisation, other services, transport, medical goods and related services (la liste des produits et prestations), biology, dental fees, therapeutic remedies and aids, drugs, medical fees |
| Detournay et al. 2017 | France | T2D patients with at least one hospitalization in the year with a main or associated diagnosis of T2D | Medical Information Systems Program: n=20,422 hospitalizations General Sample of Beneficiaries: n=484 hospitalisations | Direct | Costs for inpatient stays | Programme de médicalisation  des systèmes d’information (Medical Information Systems Program), L’Échantillon Généraliste de Bénéficiaires  (General Sample of Beneficiaries) | 2012-2014 | Inpatient costs for hospitalization for hypoglycaemia events (with and without coma), all stays and stays with hypoglycaemia as main reason |
| Detournay 2017 | France | T2D patients with antidiabetic medication | n.a. | direct, associated | Costs for antidiabetic prescriptions, overall outpatient costs, overall inpatient costs | L’Échantillon Généraliste de Bénéficiaires  (General Sample of Beneficiaries) | 2016 | Costs for antidiabetic prescriptions, overall outpatient costs, overall inpatient costs  For three groups: a) patients treated with metformin and sulphonylureas b) patients treated with metformin and dipeptidyl peptidase-4 (DPP-4) inhibitors c) patients treated with metformin and glucagon-like peptide 1 (GLP-1) agonists |
| Drabik et al. 2012 | Germany | T2DM patients ≥40 years of age and using antidiabetic medication | n=19,888 patients in a Disease-Management-Program, n=67,080 patients in regular care | direct, associated | drugs, inpatient costs | statutory health insurance (BARMER) | 2005-2007 | Average costs in the first three years in DMP,  Average costs in a three years period (not DMP),  Costs in the last year of life in DMP,  Costs in the last year of life without DMP |
| Druet et al. 2013 | France | People over 18, beneficiaries of the National Health Insurance Fund of salaried workers or the social regime of the self-employed in France having received at least three reimbursements of antidiabetic medication in the last 12 months | n=3,894 | Direct, associated | Costs for outpatient care, costs for inpatient care, costs for drugs, costs for medical devices | Survey of health insurance beneficiaries: Échantillon national témoin representative des personnes  diabétiques | 2007 | Overall healthcare costs for  outpatient care (GPs, specialists, physiotherapist, dentist, biology), medical care, drugs, transport, medical devices, others, inpatient care (public and private hospitals),  stratified for patients with and without insulin |
| Durant-Zaleski 2013 | France | T2D patients | n.a. | Direct, associated | Survey of health insurance beneficiaries: Échantillon national témoin representative des personnes  Diabétiques | Survey of health insurance beneficiaries: Échantillon national témoin representative des personnes  diabétiques | 2009 | Reimbursement for T2DM patients |
| Fritzen 2019 | Germany | Insulin treated T2D patients | n.a. | Direct, associated | Base rates at state level (hospital), statutory health insurance (AOK Hessen),  CoDiM study, market prices in 2012 | statutory health insurance (AOK Hessen),  CoDiM study | 2000-2007, 2012, 2017 | Average costs for: hypoglycaemic events (inpatient and ambulatory), costs for myocardial infarction (year of occurrence and follow-up year), costs for test strips and lancets |
| Hanaire et al. 2016 | France | T2DM patients initiating insulin therapy | n=1,233 | Direct, associated | Reimbursement of outpatient care, costs for inpatient care | Programme de médicalisation  des systèmes d’information (Medical Information Systems Program), L’Échantillon Généraliste de Bénéficiaires  (General Sample of Beneficiaries) | 2008-2013 | Overall healthcare costs 3 years before insulin initiation, 2 years before insulin initiation, 1 year before insulin initiation, 1 year after insulin initiation,  Stratified for age (<60 years, 60-75 years, ≥75 years), inpatient and ambulatory costs and initiated insulin therapy (all insulin therapies, basal insulin only) |
| Hessel et al. 2014 | Germany | T2D on multiple daily insulin administration | 32 patients (15 T1DM and 17 T2DM patients) | Direct | Costs for outpatient visits, Costs for emergency health care professional access (e.g. ambulance),  hospital costs,  Treatment costs (e.g. sugar, drinks), costs for training | Barmer GEK report, other study | 2007 | costs for severe hypoglycemic events (for patients with multiple daily insulin injections) |
| Jacob et al. 2017 | Germany | T2D, ≥40 years of age | n=36,382 (29,779 patients  treated by GPs, 6,603 patients treated by diabetologists) | Direct | human insulins and analogues, sulphonylureas, biguanides, glitazones, alpha-glucosidase inhibitors, glinides, dipeptidyl peptidase-4 (DPP-4) inhibitors, sodium-glucose cotransporter 2 (SGLT-2) inhibitors, glucagon-like peptide 1 (GLP-1) agonists | Disease Analyzer database (IMS HEALTH) | 2015 | Antihyperglycaemic treatment costs stratified by age, gender, type of health  insurance coverage, HBA1c levels, BMI, and the number of  T2DM complications |
| Jacobs et al. 2017 | Germany | T2D | 301,000 (7% of 4.3 million people  with statutory health insurance) | direct, associated | Healthcare expenses for physicians, dentists, prescribed medication, hospitals, sick benefits and other healthcare expenses, outpatient non-medical services for dialysis therapeutic remedies and aids, expenses for services abroad, inpatient benefits and rehabilitation service | routine cost data provided by  German Institute of Medical Documentation and Information | 2010 | Excess health care costs for diabetes |
| Jones et al. 2012 | Germany, France | Patients with T2D in the 6 months before and after initiation of insulin therapy | Germany n=233, France n=152 | direct | Diabetes specific costs for visits/phone calls to health care professionals; insulin; blood glucose testing strips; oral antidiabetic drugs; hospitalizations | Retrospective chart review (baseline information), prospective data collection by physician | 2006 | Total costs, costs for visits/phone calls to health care professionals; costs for insulin; costs for blood glucose testing strips; costs for oral antidiabetic drugs; costs for hospitalizations |
| Kähm et al. 2018 | Germany | T2D | n=316,220 | direct, associated | Overall costs for outpatient and inpatient services, medication, rehabilitation, and the provision of aids and appliances | statutory health insurance (Techniker Krankenkasse) | 2015 | Total healthcare costs (not diabetes specific) in the first and second year after the following complications:  angina pectoris, chronic heart failure, nonfatal and fatal myocardial infarction /cardiac arrest, nonfatal and fatal stroke, nonfatal and fatal other ischemic heart diseases, retinopathy, blindness, diabetic foot, lower-extremity amputation, nephropathy, end-stage renal disease,  patients without any complication |
| Liebl et al. 2012 | Germany | adults with T2D and insulin initiation as part of usual care in the diabetological practice because of none-adequate glycemic control under exhausted oral antidiabetic therapy according to the treating physician | n=153 | direct | Costs for physician visits and telephone calls with medical staff, Costs for OAD, Costs for insulin, Costs for self-measurement of blood glucose and Costs for hospitalization because of diabetes-related complications | Physician/ investigator assessing information from patients, costs: EBM, DRG, average pharmacy retail price | 2006 | Costs in the time period 6 month for + 6 months after insulin initiation and the year after insulin initiation for:  visits/consultations of health care professionals,  phone calls with health care professionals, blood glucose measurement, oral antidiabetic drugs,  insulin,  hospitalisation,  total costs |
| Liebl et al. 2014 | Germany | T2D on insulin aspart or regular insulin | n.a.  (decision analysis model) | associated | insulin costs plus costs for complications | Disease Analyzer database, other studies | 2010 | Total costs for the following complications: myocardial infarction, stroke or transient ischaemic attack, peripheral vascular disease, coronary heart disease  For patients with insulin aspart  For patients with regular insulin |
| McDonell et al. 2015 | Germany | T2D, initiating exenatide BID or liraglutide | n=22,232 exenatide BID, n=19,395 liraglutide | direct | costs for exenatide and liraglutide | LRx (longitudinal database that records details of prescriptions dispensed in pharmacies) | 04/2007 – 05/2011 | costs for Exenatide BID twice daily, costs for Liraglutide |
| Müller et al. 2015 | Germany | T2D | n=2,700,000 | direct, associated | overall healthcare inpatient and outpatient costs | statutory health insurance (AOK) | 2010 | Total healthcare costs (not diabetes related) |
| Sittig et al. 2015 | Germany | employed T2D | n=2,379 | direct, associated | Overall healthcare inpatient care, outpatient care and drug prescriptions costs | statutory health insurance (insurance company not mentioned) | 2007 | Total healthcare costs (not diabetes related) for:  Inpatient care, drug prescription, outpatient care, total costs |
| Ulrich et al. 2016 | Germany | T2D diagnosed at 40 years of age or later | n=880 | direct, indirect, associated | Costs for outpatient services, hospital care, rehabilitation, medication, indirect costs (long-term incapacity of work) | population based survey (KORA surveys) | 2014 | Excess costs for: total direct healthcare costs, costs for physician visits (GPs, specialists, others, total), hospital inpatient costs, hospital outpatient costs, rehabilitation outpatient costs, rehabilitation inpatient costs, medication, indirect costs, inability to work costs  Total excess costs stratified for: cardiovascular complications, antidiabetic treatment (no drugs, OAD only, OAD+insulin, insulin only), HbA1c (<6.5%, ≤6.5%-<7.5%, ≤7.5% - <9%, ≥9%), disease duration (0-2 years, 3-10 years, 11-19 years, ≥20 years) |
| Waldeyer et al. 2013 | Germany | T2D, ≥ 40 years of age | n.a.  (time-dependent illness-death model) | Direct, associated | costs for treatment, prevention, rehabilitation and  care directly related to the consumption of monetary  resources | Projection of the Federal Statistical Office of Germany, statutory health insurance (insurance company not mentioned), other studies | 2010 | Excess healthcare costs for 2010, for 2020, for 2030 for 2040 |
| Wilke et al. 2016 | Germany | T2D | n=456,586 | direct, associated | Urinary tract infection related costs | statutory health insurance (AOK PLUS) | 2010-2012 | Excess medical costs for T2DM patients with at least one urinary tract infection episode |

Table 4: Results of the quality assessment of the included papers

| **Reference** | **Category** | | | | | | | | | | **QA Score** |
| --- | --- | --- | --- | --- | --- | --- | --- | --- | --- | --- | --- |
|  | **1** | **2** | **3** | **4** | **5** | **6** | **7** | **8** | **9** | **10** |  |
| Anderten et al. 2015 | yes | yes | yes | partial | yes | partial | yes | yes | partial | yes | **8,5** |
| Baudot 2019 | yes | yes | yes | yes | yes | yes | yes | yes | no | yes | **9** |
| Charbonnel 2018 | yes | yes | yes | yes | partial | yes | yes | yes | no | yes | **8,5** |
| Detournay et al. 2015 | yes | partial | partial | yes | yes | partial | yes | yes | no | yes | **7,5** |
| Detournay et al. 2017 | yes | partial | partial | yes | yes | partial | yes | yes | no | yes | **7,5** |
| Detournay 2017 | yes | partial | partial | yes | yes | partial | yes | yes | no | yes | **7,5** |
| Drabik et al. 2012 | partial | yes | no | yes | yes | partial | yes | yes | yes | yes | **8** |
| Druet et al. 2013 | yes | yes | partial | yes | yes | partial | yes | yes | no | yes | **8** |
| Durant-Zaleski 2013 | yes | yes | partial | yes | yes | partial | yes | yes | no | yes | **8** |
| Fritzen 2019 | no | yes | no | yes | yes | partial | partial | yes | no | yes | **5** |
| Hanaire et al. 2016 | yes | yes | partial | partial | yes | yes | yes | yes | no | yes | **8** |
| Hessel et al. 2014 | partial | yes | yes | yes | yes | yes | partial | yes | no | yes | **8** |
| Jacob et al. 2017 | partial | yes | partial | partial | yes | partial | yes | yes | no | yes | **7** |
| Jacobs et al. 2017 | yes | yes | yes | yes | yes | yes | yes | yes | no | yes | **9** |
| Jones et al. 2012 | yes | yes | yes | yes | partial | yes | yes | partial | no | yes | **8** |
| Kähm et al. 2018 | yes | yes | partial | partial | yes | no | yes | partial | yes | yes | **6** |
| Liebl et al. 2012 | yes | yes | yes | yes | yes | yes | yes | yes | no | yes | **9** |
| Liebl et al. 2014 | partial | yes | yes | yes | partial | yes | yes | yes | yes | yes | **9** |
| McDonell et al. 2015 | yes | yes | yes | no | no | partial | yes | yes | no | yes | **6,5** |
| Müller et al. 2015 | yes | yes | no | yes | no | no | no | no | no | yes | **4** |
| Sittig et al. 2015 | yes | partial | no | partial | yes | no | yes | yes | no | partial | **5,5** |
| Ulrich et al. 2016 | yes | partial | yes | yes | yes | yes | yes | partial | yes | partial | **8,5** |
| Waldeyer et al. 2013 | yes | yes | partial | yes | partial | yes | yes | yes | yes | partial | **8,5** |
| Wilke et al. 2016 | partial | yes | partial | yes | yes | yes | yes | yes | no | yes | **8** |

QA score was calculated as: yes (fully met) = 1 point; partial (partially met) = 0,5 points; no (not met) = 0 points.

| Categories of the quality assurance: | |
| --- | --- |
| 1 | Was a clear definition of the illness (diabetes type 2) given? |
| 2 | Were epidemiological sources carefully described? |
| 3 | Were direct/indirect costs sufficiently disaggregated? |
| 4 | Were activity data sources carefully described? |
| 5 | Were activity data appropriately assessed? |
| 6 | Were the sources of all cost values analytically described? |
| 7 | Were unit costs appropriately valued? |
| 8 | Were the methods adopted carefully explained? |
| 9 | Were the major assumptions tested in a sensitivity analysis? |
| 10 | Was the presentation of results consistent with the methodology of the study? |

Tables 5-20: Direct and indirect costs of diabetes in Germany as presented in the studies meeting the pre-defined inclusion/exclusion criteria.

^1^ direct costs, overall healthcare;

^2^ direct costs referring to diabetes-specific treatments;

^3^ direct costs referring to the diabetes-associated treatments of complications;

^4^ indirect costs;

^*^ overall direct cost items presented in the Box and Whisker plot (Figure 2)

Table 5: Data for Germany from Anderten et al. 2015

| **Anderten et al. 2015** | | |
| --- | --- | --- |
| Patient population | T2D, initiation of insulin glargine or neutral protamine hagedorn insulin | |
| Time period | 01/2008 - 03/2014 | |
| Source of costs | Disease Analyser database (IMS HEALTH), Lauer Taxe/ EBM | |
| Reported cost category | | Annual costs per patient (€) |
| diabetes-specific direct costs: prior insulin initiation (glargine) for patients persisting with this treatment at least 3 months^2,*^ | | 843.07 |
| diabetes-specific direct costs: post insulin initiation (glargine) for patients persisting with this treatment at least 3 months^2,*^ | | 1286.68 |
| diabetes-specific direct costs: prior insulin initiation (glargine) for patients discontinuing this treatment within 3 months^2,*^ | | 774.66 |
| diabetes-specific direct costs: post insulin initiation (glargine) for patients discontinuing this treatment within 3 months^2,*^ | | 1323.10 |
| diabetes-specific direct costs: prior insulin initiation (NPH insulin) for patients persisting with this treatment at least 3 months^2,*^ | | 757.00 |
| diabetes-specific direct costs: post insulin initiation (NPH insulin) for patients persisting with this treatment at least 3 months^2,*^ | | 1280.06 |
| diabetes-specific direct costs: prior insulin initiation (NPH insulin) for patients discontinuing this treatment within 3 months^2,*^ | | 798.93 |
| diabetes-specific direct costs: post insulin initiation (NPH insulin) for patients discontinuing this treatment within 3 months^2,*^ | | 1277.85 |

Table 6: Data for Germany from Drabik et al. 2012

| **Drabik et al. 2012** | | |
| --- | --- | --- |
| Patient population | T2D, ≥40 years of age and using antidiabetic medication | |
| Time period | 2005-2007 | |
| Source of costs | statutory health insurance (BARMER) | |
| Reported cost category | | Annual costs per patient (€) |
| overall direct healthcare costs: average for the first three years of enrolment in a T2D disease management program^1,*^ | | 3973.15 |
| overall direct healthcare costs: average of a three year period for patients not enrolled in a T2D disease management program^1,*^ | | 4274.27 |
| overall direct healthcare costs: last year of life for patients enrolled in a T2D disease management program^1,*^ | | 20249.61 |
| overall direct healthcare costs: last year of life for patients not enrolled in a T2D disease management program^1,*^ | | 18874.05 |

Table 7: Data for Germany from Fritzen et al. 2019

| **Fritzen et al. 2019** | | |
| --- | --- | --- |
| Patient population | insulin treated T2D patients | |
| Time period | 2000-2007, 2012, 2017 | |
| Source of costs | Base rates at state level (hospital), statutory health insurance (AOK Hessen), CoDiM study, market prices in 2012 | |
| Reported cost category | | Annual costs per patient (€) |
| diabetes-associated direct costs: hypoglycaemic episodes (ambulatory)^3,*^ | | 531.96 |
| diabetes-associated direct costs: hypoglycaemic episodes (Hospitalisation)^3,*^ | | 2434.74 |
| diabetes-associated direct costs: myocardial infarction (year of occurrence)^3,*^ | | 12448.04 |
| diabetes-associated direct costs: myocardial infarctions (Follow-up, first year)^3,*^ | | 5138.78 |
| diabetes-specific direct costs: test strips^2^ | | 0.61 |
| diabetes-specific direct costs: lancets^2^ | | 0.08 |

Table 8: Data for Germany from Hessel et al. 2014

| **Hessel et al. 2014** | | |
| --- | --- | --- |
| Patient population | T2D on multiple daily insulin administration | |
| Time period | 2007 | |
| Source of costs | Barmer GEK report, other study | |
| Reported cost category | | Annual costs per patient (€) |
| diabetes-associated direct costs: severe hypoglycaemic events (~0,1 severe hypoglycaemic events per patient per year)^3,*^ | | 98.91 |

Table 9: Data for Germany from Jacob et al. 2017

| **Jacob et al. 2017** | | |
| --- | --- | --- |
| Patient population | T2D, ≥40 years of age | |
| Time period | 2015 | |
| Source of costs | Disease Analyzer database (IMS HEALTH) | |
| Reported cost category | | Annual costs per patient (€) |
| diabetes-specific direct cost: antihyperglycemic treatment^2^ | | 516.28 |
| diabetes-specific direct cost: antihyperglycemic treatment (women)^2^ | | 491.40 |
| diabetes-specific direct cost: antihyperglycemic treatment (men)^2^ | | 537.01 |
| diabetes-specific direct cost: antihyperglycemic treatment for patients ≤60 years^2^ | | 568.11 |
| diabetes-specific direct cost: antihyperglycemic treatment for patients 61 to 70 years^2^ | | 567.07 |
| diabetes-specific direct cost: antihyperglycemic treatment for patients 71 to 80 years^2^ | | 483.10 |
| diabetes-specific direct cost: antihyperglycemic treatment for patients >80 years^2^ | | 402.24 |
| diabetes-specific direct cost: antihyperglycemic treatment for patients with HbA1c <6.5^2^ | | 288.20 |
| diabetes-specific cost: antihyperglycemic treatment for patients  with HbA1c 6.5 to <7.5^2^ | | 484.14 |
| diabetes-specific direct cost: antihyperglycemic treatment for patients with HbA1c 7.5 to <9^2^ | | 793.08 |
| diabetes-specific direct cost: antihyperglycemic treatment for patients with HbA1c ≥9^2^ | | 882.23 |
| diabetes-specific direct cost: antihyperglycemic treatment for patients with BMI <30^2^ | | 405.35 |
| diabetes-specific direct cost: antihyperglycemic treatment for patients with BMI 30-35^2^ | | 528.72 |
| diabetes-specific direct cost: antihyperglycemic treatment for patients with BMI ≥35^2^ | | 718.43 |
| diabetes-specific direct cost: antihyperglycemic treatment for patients without complications^2^ | | 403.28 |
| diabetes-specific direct cost: antihyperglycemic treatment for patients with 1 complication^2^ | | 482.07 |
| diabetes-specific direct cost: antihyperglycemic treatment for patients with 2 complications^2^ | | 579.52 |
| diabetes-specific direct cost: antihyperglycemic treatment for patients with 3 complications^2^ | | 644.83 |
| diabetes-specific direct cost: antihyperglycemic treatment for patients with >3 complications^2^ | | 780.64 |

Table 10: Data for Germany from Jacobs et al. 2017

| **Jacobs et al. 2017** | | |
| --- | --- | --- |
| Patient population | T2D | |
| Time period | 2010 | |
| Source of costs | routine cost data provided by German Institute of Medical Documentation and Information | |
| Reported cost category | | Annual costs per patient (€) |
| overall direct healthcare costs (excess costs)^3,*^ | | 3558.13 |

Table 11: Data for Germany from Jones et al. 2012

| **Jones et al. 2012** | | |
| --- | --- | --- |
| Patient population | Patients with T2D in the 6 months before and after initiation of insulin therapy | |
| Time period | 2006 | |
| Source of costs | Retrospective chart review (baseline information), prospective data collection by physician | |
| Reported cost category | | Annual costs per patient (€) |
| diabetes-specific direct costs: consultation with general practice professionals (visits and phone calls)^2^ | | 204.76 |
| diabetes-specific direct costs: specialist care for glycaemic control^2^ | | 298.15 |
| diabetes-specific direct costs: oral antidiabetic drugs^2^ | | 196.37 |
| diabetes-specific direct costs: insulin^2^ | | 274.20 |
| diabetes-specific direct cost: blood glucose monitoring^2^ | | 638.21 |
| diabetes-specific direct costs: hospitalisation^2^ | | 176.02 |
| diabetes-specific direct costs: other diabetes related treatment (visits/calls to dieticians, ophthalmologists, chiropodists, podiatrists)^2^ | | 59.87 |
| diabetes-specific direct costs for diabetes treatment (total)^2,*^ | | 1844.00 |

Table 12: Data for Germany from Kähm et al. 2018

| **Kähm et al. 2018** | | |
| --- | --- | --- |
| Patient population | T2D | |
| Time period | 2015 | |
| Source of costs | statutory health insurance (Techniker Krankenkasse) | |
| Reported cost category | | Annual costs per patient (€) |
| overall direct healthcare costs: T2D patients, no complication^1,*^ | | 2793.33 |
| overall direct healthcare costs: T2D patients with foot complications, year of the first documentation of a specific code for foot complications^1,*^ | | 3997.84 |
| overall direct healthcare costs: T2D patients with foot complications, second year after the first documentation of a specific code for foot complications^1.*^ | | 4263.68 |
| overall direct healthcare costs: T2D patients with lower extremity amputation, year of amputation^1,*^ | | 20512.96 |
| overall direct healthcare costs: T2D patients with lower extremity amputation, second year after amputation^1,*^ | | 12818.02 |
| overall direct healthcare costs: T2D patients with retinopathy, year of the first documentation of a specific code for retinopathy^1,*^ | | 2862.63 |
| overall direct healthcare costs: T2D patients with retinopathy, second year after the first documentation of a specific code for retinopathy^1,*^ | | 2923.02 |
| overall direct healthcare costs: T2D patients with blindness, year of becoming blind^1,*^ | | 5754.92 |
| overall direct healthcare costs: T2D patients with blindness, second year after becoming blind^1,*^ | | 4496.14 |
| overall direct healthcare costs: T2D patients with nephropathy, year of the first documentation of a specific code for nephropathy^1,*^ | | 6379.53 |
| overall direct healthcare costs: T2D patients with nephropathy, second year after the first documentation of a specific code for nephropathy^1,*^ | | 5264.42 |
| overall direct healthcare costs: T2D patients with end-stage renal disease, year of the first documentation of a specific code for end-stage renal disease^1.*^ | | 32738.14 |
| overall direct healthcare costs: T2D patients with end-stage renal disease, second year after the first documentation of a specific code for end-stage renal disease^1,*^ | | 23629.17 |
| overall direct healthcare costs: T2D patients with nonfatal stroke, year of this stroke^1,*^ | | 15127.37 |
| overall direct healthcare costs: T2D patients with nonfatal stroke, second year after this stroke^1,^* | | 10549.15 |
| overall direct healthcare costs: T2D patients with fatal stroke, year of this fatal stroke^1,*^ | | 12804.70 |
| overall direct healthcare costs: T2D patients with nonfatal myocardial infarction, year of this nonfatal myocardial infarction^1,*^ | | 11126.25 |
| overall direct healthcare costs: T2D patients with nonfatal myocardial infarction, second year after the nonfatal myocardial infarction^1,*^ | | 5263.87 |
| overall direct healthcare costs: T2D patients with fatal myocardial infarction, year of the fatal myocardial infarction^1,*^ | | 9537.64 |
| overall direct healthcare costs: T2D patients with nonfatal ischemic heart disease, year of the nonfatal myocardial infarction^1,*^ | | 9361.57 |
| overall direct healthcare costs: T2D patients with nonfatal ischemic heart disease, second year after the nonfatal ischemic heart disease^1,*^ | | 4402.65 |
| overall direct healthcare costs: T2D patients with fatal ischemic heart disease, year of the fatal ischemic heart disease^1,*^ | | 19874.15 |
| overall direct healthcare costs: T2D patients with angina pectoris, year of the first documentation of a specific code for angina pectoris^1,*^ | | 5070.72 |
| overall direct healthcare costs: T2D patients with angina pectoris, second year after the first documentation of a specific code for angina pectoris^1,*^ | | 3517.00 |
| overall direct healthcare costs: T2D patients with chronic heart failure, year of the first documentation of a specific code for chronic heart failure^1,*^ | | 7315.96 |
| overall direct healthcare costs: T2D patients with chronic heart failure, second year after the first documentation of a specific code for chronic heart failure^1,*^ | | 5958.19 |

Table 13: Data for Germany from Liebl et al. 2012

| **Liebl et al. 2012** | | |
| --- | --- | --- |
| Patient population | adults with T2D and insulin initiation as part of usual care in the diabetological practice because of none-adequate glycaemic control under exhausted oral antidiabetic therapy according to the treating physician | |
| Time period | 2006 | |
| Source of costs | Physician/ investigator assessing information from patients, costs: EBM, DRG, average pharmacy retail price | |
| Reported cost category | | Annual costs per patient (€) |
| diabetes-specific direct costs: 6 months prior and after insulin initiation^2,*^ | | 1828.43 |
| diabetes-specific direct costs: visits/consultations of healthcare professionals 6 months prior and after insulin initiation^2^ | | 480.16 |
| diabetes-specific direct costs: phone calls with healthcare professionals 6 months prior and after insulin initiation^2^ | | 68.25 |
| diabetes-specific direct costs: blood glucose measurement 6 months prior and after insulin initiation^2^ | | 644.20 |
| diabetes-specific direct costs: OAD 6 months prior and after insulin initiation^2^ | | 198.77 |
| diabetes-specific direct costs: insulin 6 months prior and after insulin initiation^2^ | | 296.96 |
| diabetes-associated direct costs: hospitalisations 6 months prior and after insulin initiation^2^ | | 141.29 |
| diabetes-related direct costs: 1 year after insulin initiation^2,*^ | | 2204.41 |
| diabetes-specific direct costs: visits/consultations of healthcare professionals 1 year after insulin initiation^2^ | | 387.96 |
| diabetes-specific direct costs: phone calls with healthcare professionals 1 year after insulin initiation^2^ | | 50.29 |
| diabetes-specific direct costs: blood glucose measurement 1 year after insulin initiation^2^ | | 943.55 |
| diabetes-specific direct costs: OAD 1 year after insulin initiation^2^ | | 95.79 |
| diabetes-specific direct costs: insulin 1 year after insulin initiation^2^ | | 644.20 |
| diabetes-specific direct costs: hospitalisations 1 year after insulin initiation^2^ | | 83.82 |

Table 14: Data for Germany from Liebl et al. 2014

| **Liebl et al. 2014** | | |
| --- | --- | --- |
| Patient population | T2D on insulin aspart or regular insulin | |
| Time period | 2010 | |
| Source of costs | other studies | |
| Reported cost category | | Annual costs per patient (€) |
| diabetes-associated direct costs: myocardial infarction, stroke or transient ischaemic attack, peripheral vascular disease, coronary heart disease for patients on regular insulin^3,*^ | | 2220.76 |
| diabetes-associated direct costs: myocardial infarction, stroke or transient ischaemic attack, peripheral vascular disease, coronary heart disease for patients on insulin aspart^3,*^ | | 1642.24 |

Table 15: Data for Germany from McDonell et al. 2015

| **McDonell et al. 2015** | | |
| --- | --- | --- |
| Patient population | T2D, initiating exenatide BID or liraglutide | |
| Time period | 04/2007 - 05/2011 | |
| Source of costs | LRx (longitudinal database that records details of prescriptions dispensed in pharmacies) | |
| Reported cost category | | Annual costs per patient (€) |
| diabetes-specific direct costs for exenatide BID twice daily^2^ | | 1671.11 |
| diabetes-specific direct costs for liraglutide^2^ | | 1887.27 |

Table 16: Data for Germany from Müller et al. 2015

| **Müller et al. 2015** | | |
| --- | --- | --- |
| Patient population | T2D | |
| Time period | 2010 | |
| Source of costs | statutory health insurance (AOK) | |
| Reported cost category | | Annual costs per patient (€) |
| overall direct healthcare costs^1,*^ | | 4882.11 |

Table 17: Data for Germany from Sittig et al. 2015

| **Sittig et al. 2015** | | |
| --- | --- | --- |
| Patient population | employed T2D | |
| Time period | 2007 | |
| Source of costs | statutory health insurance (insurance company not mentioned) | |
| Reported cost category | | Annual costs per patient (€) |
| overall direct healthcare costs^1,*^ | | 3081.11 |
| overall direct healthcare costs: inpatient care^1^ | | 1142.20 |
| overall direct healthcare costs: drugs^1^ | | 1172.75 |
| overall direct healthcare costs: outpatient care^1^ | | 766.17 |

Table 18: Data for Germany from Ulrich et al. 2016

| **Ulrich et al. 2016** | | |
| --- | --- | --- |
| Patient population | T2D diagnosed at 40 years of age or later | |
| Time period | 2014 | |
| Source of costs | population based survey (KORA surveys) | |
| Reported cost category | | Annual costs per patient (€) |
| overall direct healthcare costs^1.*^ | | 3482.73 |
| overall direct healthcare costs: GP visits^1^ | | 141.30 |
| overall direct healthcare costs: specialists visits^1^ | | 94.55 |
| overall direct healthcare costs: other physician visits^1^ | | 221.31 |
| overall direct healthcare costs: all physician visits^1^ | | 462.36 |
| overall direct healthcare costs: hospital inpatient^1^ | | 1728.90 |
| overall direct healthcare costs: hospital outpatient^1^ | | 19.74 |
| overall direct healthcare costs: inpatient rehabilitation^1^ | | 188.06 |
| overall direct healthcare costs: outpatient rehabilitation^1^ | | 14.55 |
| overall direct healthcare costs: medication^1^ | | 997.44 |
| indirect costs^4^ | | 4263.02 |
| Indirect costs for inability to work^4^ | | 3474.42 |
| diabetes-associated direct costs: overall direct healthcare costs, excess costs^3,*^ | | 1561.62 |
| diabetes-associated direct costs: overall healthcare direct costs for GP visits, excess costs^3^ | | 50.91 |
| diabetes-associated direct costs: overall healthcare direct costs for specialists visits, excess costs^3^ | | 50.91 |
| diabetes-associated direct costs: overall healthcare direct costs for other physician visits, excess costs^3^ | | 58.18 |
| diabetes-associated direct costs: overall healthcare direct costs for all physician visits, excess costs^3^ | | 161.05 |
| diabetes-associated direct costs: overall healthcare direct costs for hospital inpatient, excess costs^3^ | | 776.13 |
| diabetes-associated direct costs: overall healthcare direct costs for hospital outpatient, excess costs^3^ | | -2.08 |
| diabetes-associated direct costs: overall healthcare direct costs for inpatient rehabilitation, excess costs^3^ | | 84.16 |
| diabetes-associated direct costs: overall healthcare direct costs for outpatient rehabilitation, excess costs^3^ | | -7.27 |
| diabetes-associated direct costs: overall healthcare direct costs for medication, excess costs^3^ | | 500.80 |
| indirect costs, excess costs^4^ | | 2204.76 |
| indirect costs: inability to work, excess costs^4^ | | 2124.76 |
| overall healthcare direct costs: patients without cardiovascular complications, excess costs^3,*^ | | 1037.40 |
| diabetes-associated direct costs: overall healthcare direct costs, patients with cardiovascular complications, excess costs^3,*^ | | 3400.37 |
| diabetes-associated direct costs: overall healthcare direct costs, patients without pharmacological treatment, excess costs^3,*^ | | 499.49 |
| diabetes-associated direct costs: overall healthcare direct costs, patients on OAD only, excess costs^3,*^ | | 1018.19 |
| diabetes-associated direct costs: overall healthcare direct costs, patients on OAD + insulin, excess costs^3,*^ | | 3035.36 |
| diabetes-associated direct costs: overall healthcare direct costs, patients on insulin only, excess costs^3,*^ | | 5724.91 |
| diabetes-associated direct costs: overall healthcare direct costs, patients with HbA1c <6.5 %, excess costs^3,*^ | | 1671.37 |
| diabetes-associated direct costs: overall healthcare direct costs, patients with 6.5 ≤ HbA1c <7.5%, excess costs^3,*^ | | 1229.51 |
| diabetes-associated direct costs: overall healthcare direct costs patients with 7.5 ≤ HbA1c <9.0%, excess costs^3,*^ | | 1805.84 |
| diabetes-associated direct costs: overall healthcare direct costs, patients with HbA1c ≥9.0%, excess costs^3,*^ | | 2382.18 |
| diabetes-associated direct costs: overall healthcare direct costs, patients with disease duration 0-2 years, excess costs^3,*^ | | 1729.00 |
| diabetes-associated direct costs: overall healthcare direct costs: patients with disease duration 3-10 years, excess costs^3,*^ | | 845.29 |
| diabetes-associated direct costs: overall healthcare direct costs, patients with disease duration 11-19 years, excess costs^3,*^ | | 2324.54 |
| diabetes-associated direct costs: overall healthcare direct costs, patients with disease duration ≥ 20 years, excess costs^3,*^ | | 3880.64 |

Table 19: Data for Germany from Waldeyer et al. 2013

| **Waldeyer et al. 2013** | | |
| --- | --- | --- |
| Patient population | T2D, ≥40 years of age | |
| Time period | 2010 | |
| Source of costs | Projection of the Federal Statistical Office of Germany, statutory health insurance (insurance company not mentioned), other studies | |
| Reported cost category | | Annual costs per patient (€) |
| diabetes-associated direct costs: overall direct healthcare costs patients in 2010, excess costs^3^ | | 3796.34 |
| diabetes-associated direct costs: overall direct healthcare costs patients in 2020, excess costs | | 4097.39 |
| diabetes-associated direct costs: overall direct healthcare costs patients in 2030, excess costs | | 4319.92 |
| diabetes-associated direct costs: overall direct healthcare costs patients in 2040, excess costs | | 4357.73 |

Table 20: Data for Germany from Wilke et al. 2016

| **Wilke et al. 2016** | | |
| --- | --- | --- |
| Patient population | T2D | |
| Time period | 2010-2012 | |
| Source of costs | statutory health insurance (AOK PLUS) | |
| Reported cost category | | Annual costs per patient (€) |
| excess medical costs for T2D patients with at least one urinary tract infection episode compared to patients without urinary tract infection^3,*^ | | 4253.26 |

Tables 21-29: Direct and indirect costs of diabetes in France as presented in the studies meeting the pre-defined inclusion/exclusion criteria.

^1^ direct costs, overall healthcare;

^2^ direct costs referring to diabetes-specific treatments;

^3^ direct costs referring to the diabetes-associated treatments of complications;

^4^ indirect costs;

^*^ overall direct cost items presented in the Box and Whisker plot (Figure 2)

Table 21: Data for France from Baudot et al. 2019

| **Baudot et al. 2019** | | |
| --- | --- | --- |
| Patient population | T2D, ≥ 45 years of age and on antidiabetic medication, incident patients in 2008 | |
| Time period | 2015 | |
| Source of costs | National health insurance administrative database | |
| Reported cost category | | Annual costs per patient (€) |
| overall direct healthcare costs: primary care^1^ | | 3186.82 |
| overall direct healthcare costs: hospital care^1^ | | 2049.56 |
| overall direct healthcare costs: cash benefits^1^ | | 472.74 |
| overall direct healthcare costs^1,*^ | | 5709.11 |
| diabetes-associated direct costs: overall healthcare direct costs, primary care, excess costs^3^ | | 1306.24 |
| diabetes-associated direct costs: overall healthcare direct costs, hospital care, excess costs^3^ | | 549.45 |
| diabetes-associated direct costs: overall healthcare direct costs, cash benefits, excess costs^3^ | | 101.60 |
| diabetes-associated direct costs: overall healthcare direct costs, excess costs^3,*^ | | 1958.33 |
| overall direct healthcare costs: nursing care^1^ | | 550.49 |
| overall direct healthcare costs: other paramedical care^1^ | | 14.51 |
| overall direct healthcare costs: drugs^1^ | | 1251.30 |
| overall direct healthcare costs: medical devices and services^1^ | | 378.40 |
| overall direct healthcare costs: general practitioner^1^ | | 214.60 |
| overall direct healthcare costs: other specialist care^1^ | | 235.33 |
| overall direct healthcare costs: physiotherapy^1^ | | 129.58 |
| overall direct healthcare costs: clinical pathology^1^ | | 132.70 |
| overall direct healthcare costs: transportation^1^ | | 217.71 |
| overall direct healthcare costs: other primary care^1^ | | 10.37 |

Table 22: Data for France from Charbonnel et al. 2018

| **Charbonnel et al. 2018** | | |
| --- | --- | --- |
| Patient population | T2D patients on antidiabetic medication | |
| Time period | 2013 | |
| Source of costs | L’Échantillon Généraliste de Bénéficiaires (General Sample of Beneficiaries) | |
| Reported cost category | | Annual costs per patient (€) |
| overall direct healthcare costs^1,*^ | | 6793.57 |
| overall direct healthcare costs. ambulatory costs^1^ | | 4539.14 |
| overall direct healthcare costs: hospital costs^1^ | | 2254.43 |
| overall direct healthcare costs: medication^1^ | | 1609.11 |
| overall direct healthcare costs: physician consultation^1^ | | 243.30 |
| overall direct healthcare costs: home visits^1^ | | 60.56 |
| overall direct healthcare costs: interventions^1^ | | 333.10 |
| overall direct healthcare costs: nursing care^1^ | | 743.47 |
| overall direct healthcare costs: physiotherapy^1^ | | 156.63 |
| overall direct healthcare costs: medical devices^1^ | | 608.77 |
| overall direct healthcare costs: laboratory tests^1^ | | 209.88 |
| overall direct healthcare costs: transportation^1^ | | 246.43 |
| diabetes-associated direct costs: overall healthcare direct costs, excess costs^3,*^ | | 2963.44 |
| diabetes-associated direct costs: overall healthcare direct costs, ambulatory cost, excess costs^3^ | | 2070.65 |
| diabetes-associated direct costs: overall healthcare direct costs, hospital costs, excess costs^3^ | | 892.79 |
| diabetes-associated direct costs: overall healthcare direct costs, medication, excess costs^3^ | | 845.80 |
| diabetes-associated direct costs: overall healthcare direct costs, physician consultation, excess costs^3^ | | 43.86 |
| diabetes-associated direct costs: overall healthcare direct costs, home visits, excess costs^3^ | | 26.11 |
| diabetes-associated direct costs: overall healthcare direct costs, interventions, excess costs^3^ | | 45.95 |
| diabetes-associated direct costs: overall healthcare direct costs, for nursing care, excess costs^3^ | | 553.43 |
| diabetes-associated direct costs: overall healthcare direct costs, physiotherapy, excess costs^3^ | | 29.24 |
| diabetes-associated direct costs: overall healthcare direct costs, medical devices, excess costs^3^ | | 286.11 |
| diabetes-associated direct costs: overall healthcare direct costs, laboratory tests, excess costs^3^ | | 85,62 |
| diabetes-associated direct costs: overall healthcare direct costs, transportation, excess costs^3^ | | 134.70 |

Table 23: Data for France from Detournay 2015

| **Detournay 2015** | | |
| --- | --- | --- |
| Patient population | T2D patients treated with insulin over one year (excluding incident cases, cases that died in that year, cases released during that year from the base, patients reporting to local mutualist) | |
| Time period | 2013 | |
| Source of costs | L’Échantillon Généraliste de Bénéficiaires (General Sample of Beneficiaries) | |
| Reported cost category | | Annual costs per patient (€) |
| overall direct healthcare costs^1,*^ | | 13061.90 |
| diabetes-associated direct costs: overall healthcare direct costs, excess costs^3,*^ | | 4050.45 |
| overall direct healthcare costs: hospitalisation^1^ | | 3882.34 |
| overall direct healthcare costs: physician consultation^1^ | | 1000.34 |
| overall direct healthcare costs: medical devices and interventions^1^ | | 1435.78 |
| overall direct healthcare costs: paramedical services (e.g. nursing care)^1^ | | 2935.25 |
| overall direct healthcare costs: transport^1^ | | 567.00 |
| overall direct healthcare costs: laboratory tests^1^ | | 282.98 |
| overall direct healthcare costs: drugs^1^ | | 2798.46 |
| diabetes-associated direct costs: overall direct healthcare costs, hospitalisation, excess costs^3^ | | 1409.67 |
| diabetes-associated direct costs: overall direct healthcare costs, paramedical services (e.g. nursing care), excess costs^3^ | | 393.66 |
| diabetes-associated direct costs: overall healthcare direct costs, drugs, excess costs^3^ | | 808.21 |
| diabetes-associated direct costs: overall healthcare direct costs, physician consultation, excess costs^3^ | | 621.30 |

Table 24: Data for France from Detournay 2017

| **Detournay 2017** | | | |
| --- | --- | --- | --- |
| Patient population | T2D patients with antidiabetic medication | |  |
| Time period | 2016 | |  |
| Source of costs | L’Échantillon Généraliste de Bénéficiaires (General Sample of Beneficiaries) | |  |
| Reported cost category | | Annual costs per patient (€) |  |
| overall healthcare direct costs: patients on metformin plus sulfonylurea^1,*^ | | 3717.22 |  |
| overall healthcare direct costs: patients on metformin plus DDP4 inhibtor^1,*^ | | 4204.62 |  |
| overall healthcare direct costs: patients om metformin plus GLP-1 receptor agonist^1,*^ | | 7906.82 |  |
| overall healthcare direct costs: ambulatory care, patients on metformin plus sulfonylurea^1^ | | 2303,53 |  |
| overall healthcare direct costs: ambulatory care, patients on metformin plus DDP4 inhibitor^1^ | | 2539.30 |  |
| overall healthcare direct costs: ambulatory care, patients on metformin plus GLP-1 receptor agonist^1^ | | 5365.65 |  |
| overall healthcare direct costs: hospital care, patients on metformin plus sulfonylurea^1^ | | 940.49 |  |
| overall healthcare direct costs: hospital care, patients on metformin plus DDP4 inhibitor^1^ | | 1147.78 |  |
| overall healthcare direct costs: hospital care, patients on metformin plus GLP-1 receptor agonist^1^ | | 1814.48 |  |
| overall healthcare direct costs: medication, patients on metformin plus sulfonylurea^1^ | | 162.84 |  |
| overall healthcare direct costs: medication, patients on metformin plus DDP4 inhibitor^1^ | | 457.97 |  |
| overall healthcare direct costs: medication, patients on metformin plus GLP-1 receptor agonist^1^ | | 1498.53 |  |

Table 25: Data for France from Detournay et al. 2017

| **Detournay et al. 2017** | | |
| --- | --- | --- |
| Patient population | T2D patients with at least one hospitalisation in the year with a main or associated diagnosis of T2D | |
| Time period | 2012 - 2014 | |
| Source of costs | Programme de médicalisation des systèmes d’information (Medical Information Systems Program), L’Échantillon Généraliste de Bénéficiaires (General Sample of Beneficiaries) | |
| Reported cost category | | Annual costs per patient (€) |
| diabetes-associated direct costs: hospitalisation for any stay with a hypoglycaemia diagnosis (without diabetic coma)^3^ | | 5241.13 |
| diabetes-specific direct costs: hospitalisation for stay with hypoglycaemia as main diagnosis (without diabetic coma) ^1^ | | 3230.25 |
| diabetes-associated direct costs: hospitalisation for any stay with a hypoglycaemia diagnosis (with diabetic comas)^3^ | | 5333.19 |
| diabetes-specific direct costs: hospitalisation for any stay with hypoglycaemia as main diagnosis (with diabetic comas) ^1^ | | 3993,92 |
| diabetes-specific direct costs: hospitalisations for hypoglycaemia (EGB data base)^1^ | | 4926.54 |

Table 26: Data for France from Druet et al. 2013

| **Druet et al. 2013** | | |
| --- | --- | --- |
| Patient population | People over 18, beneficiaries of the National Health Insurance Fund of salaried workers or the social regime of the self-employed in France having received at least three reimbursements of anti-diabetic medication in the last 12 months | |
| Time period | 2007 | |
| Source of costs | Survey of health insurance beneficiaries: Échantillon national témoin representative des personnes diabétiques | |
| Reported cost category | | Annual costs per patient (€) |
| overall direct healthcare costs: ambulatory care, T2D patients^1^ | | 3736.82 |
| overall direct healthcare costs: ambulatory care, T2D patients on insulin^1^ | | 7692.20 |
| overall direct healthcare costs: ambulatory care, T2D patients not on insulin^1^ | | 2830.82 |
| overall direct healthcare costs: general practitioner, T2D patients^1^ | | 265.57 |
| overall direct healthcare costs: general practitioner, T2D patients on insulin^1^ | | 345.48 |
| overall direct healthcare costs: general practitioner, T2D patients not on insulin^1^ | | 246.77 |
| overall direct healthcare costs: specialists’ consultations, T2D patients^1^ | | 235.02 |
| overall direct healthcare costs: specialists’ consultations, T2D patients on insulin^1^ | | 303.18 |
| overall direct healthcare costs: specialists’ consultations, T2D patients not on insulin^1^ | | 219.74 |
| overall direct healthcare costs: physiotherapy, T2D patients^1^ | | 102.23 |
| overall direct healthcare costs: physiotherapy, T2D patients on insulin^1^ | | 197.42 |
| overall direct healthcare costs: physiotherapy, T2D patients not on insulin^1^ | | 81.08 |
| overall direct healthcare costs: nursing care, T2D patients^1^ | | 418.34 |
| overall direct healthcare costs: nursing care, T2D patients on insulin^1^ | | 1775.58 |
| overall direct healthcare costs: nursing care, T2D patients not on insulin^1^ | | 106.93 |
| overall direct healthcare costs: laboratory testing, T2D patients^1^ | | 179.79 |
| overall direct healthcare costs: laboratory testing, T2D patients on insulin^1^ | | 231.49 |
| overall direct healthcare costs: laboratory testing, T2D patients not on insulin^1^ | | 168.04 |
| overall direct healthcare costs: drugs, T2D patients^1^ | | 1640.44 |
| overall direct healthcare costs: drugs, T2D patients on insulin^1^ | | 2719.18 |
| overall direct healthcare costs: drugs, T2D patients not on insulin^1^ | | 1392.49 |
| overall direct healthcare costs: transport, T2D patients^1^ | | 170.39 |
| overall direct healthcare costs: transport, T2D patients on insulin^1^ | | 461.81 |
| overall direct healthcare costs: transport, T2D patients not on insulin^1^ | | 103.41 |
| overall direct healthcare costs: medical devices, T2D patients^1^ | | 400.71 |
| overall direct healthcare costs: medical devices, T2D patients on insulin^1^ | | 1202.13 |
| overall direct healthcare costs: medical devices, T2D patients not on insulin^1^ | | 217.39 |
| overall direct healthcare costs: other ambulatory care, T2D patients^1^ | | 262.05 |
| overall direct healthcare costs: other ambulatory care, T2D patients on insulin^1^ | | 407.76 |
| overall direct healthcare costs: other ambulatory care, T2D patients not on insulin^1^ | | 229.14 |
| overall direct healthcare costs: hospitalisation, T2D patients^1^ | | 2009.42 |
| overall direct healthcare costs: hospitalisation, T2D patients on insulin^1^ | | 4542.94 |
| overall direct healthcare costs: hospitalisation, T2D patients not on insulin^1^ | | 1428.92 |
| overall direct healthcare costs for T2D patients^1,*^ | | 5746.24 |
| overall direct healthcare costs for T2D patients on insulin^1,*^ | | 12236.32 |
| overall direct healthcare costs for T2D patients not on insulin^1,*^ | | 4259.74 |

Table 27: Data for France from Durand-Zaleski 2013

| **Durand-Zaleski 2013** | | |
| --- | --- | --- |
| Patient population | T2D | |
| Time period | 2009 | |
| Source of costs | Survey of health insurance beneficiaries: Échantillon national témoin representative des personnes Diabétiques | |
| Reported cost category | | Annual costs per patient (€) |
| overall direct healthcare costs^1,*^ | | 4867.60 |

Table 28: Data for France from Hanaire et al. 2016

| **Hanaire et al. 2016** | | |
| --- | --- | --- |
| Patient population | T2D patients initiating insulin therapy | |
| Time period | 2008-2013 | |
| Source of costs | Programme de médicalisation des systèmes d’information (Medical Information Systems Program), L’Échantillon Généraliste de Bénéficiaires (General Sample of Beneficiaries) | |
| Reported cost category | | Annual costs per patient (€) |
| overall direct healthcare costs: 3 years before insulin initiation^1,*^ | | 5525.08 |
| overall direct healthcare costs: 3 years before insulin initiation, patients <60 years of age^1,*^ | | 7094.97 |
| overall direct healthcare costs: 3 years before insulin initiation, patients 60-75 years of age^1,*^ | | 5357.17 |
| overall direct healthcare costs: 3 years before insulin initiation, patients ≥ 75 years of age^1,*^ | | 4300.22 |
| overall direct healthcare costs: inpatient care, 3 years before insulin initiation^1^ | | 1434.17 |
| overall direct healthcare costs: ambulatory care, 3 years before insulin initiation^1^ | | 4090.91 |
| overall direct healthcare costs: transport, 3 years before insulin initiation^1^ | | 167.91 |
| overall direct healthcare costs: medical devices, 3 years before insulin initiation^1^ | | 479.59 |
| overall direct healthcare costs: laboratory tests, 3 years before insulin initiation^1^ | | 225.42 |
| overall direct healthcare costs: paramedical services, 3 years before insulin initiation^1^ | | 465.79 |
| overall direct healthcare costs: drugs, 3 years before insulin initiation^1^ | | 1776.90 |
| overall direct healthcare costs: physician consultation, 3 years before insulin initiation^1^ | | 785.52 |
| overall direct healthcare costs: 2 years before insulin initiation^1,*^ | | 5908.83 |
| overall direct healthcare costs: 2 years before insulin initiation, patients <60 years of age^1,*^ | | 7302.52 |
| overall direct healthcare costs: 2 years before insulin initiation, patients 60-75 years of age^1,*^ | | 6007.01 |
| overall direct healthcare costs: 2 years before insulin initiation, patients ≥ 75 years of age^1,*^ | | 4576.07 |
| overall direct healthcare costs: inpatient care, 2 years before insulin initiation^1^ | | 1504.29 |
| overall direct healthcare costs: ambulatory care, 2 years before insulin initiation^1^ | | 4405.66 |
| overall direct healthcare costs: transport, 2 years before insulin initiation^1^ | | 197.49 |
| overall direct healthcare costs: medical devices, 2 years before insulin initiation^1^ | | 512.34 |
| overall direct healthcare costs: laboratory tests, 2 years before insulin initiation^1^ | | 238.11 |
| overall direct healthcare costs: paramedical services, 2 years before insulin initiation^1^ | | 534.91 |
| overall direct healthcare costs: drugs, 2 years before insulin initiation^1^ | | 1989.55 |
| overall direct healthcare costs: physician consultation, 2 years before insulin initiation^1^ | | 792.21 |
| overall direct healthcare costs: 1 year before insulin initiation^1,*^ | | 7896.44 |
| overall direct healthcare costs: 1 year before insulin initiation, patients <60 years of age^1,*^ | | 10354.92 |
| overall direct healthcare costs: 1 year before insulin initiation, patients 60-75 years of age^1,*^ | | 7257.20 |
| overall direct healthcare costs: 1 year before insulin initiation, patients ≥ 75 years of age^1^ | | 6353.06 |
| overall direct healthcare costs: inpatient care, 1 year before insulin initiation^1,*^ | | 2932.43 |
| overall direct healthcare costs: ambulatory care, 1 year before insulin initiation^1^ | | 4964.01 |
| overall direct healthcare costs: transport, 1 year before insulin initiation^1^ | | 263.80 |
| overall direct healthcare costs: medical devices, 1 year before insulin initiation^1^ | | 595.46 |
| overall direct healthcare costs: laboratory tests, 1 year before insulin initiation^1^ | | 320.72 |
| overall direct healthcare costs: paramedical services, 1 year before insulin initiation^1^ | | 629.40 |
| overall direct healthcare costs: drugs, 1 year before insulin initiation^1^ | | 2014.06 |
| overall direct healthcare costs: physician consultation, 1 year before insulin initiation^1^ | | 953.40 |
| overall direct healthcare costs: 1 year after insulin initiation^1,*^ | | 12461.52 |
| overall direct healthcare costs: 1 year after insulin initiation, patients <60 years of age^1,*^ | | 15299.46 |
| overall direct healthcare costs: 1 year after insulin initiation, patients 60-75 years of age^1,*^ | | 12677.28 |
| overall direct healthcare costs: 1 year after insulin initiation, patients ≥ 75 years of age^1,*^ | | 9728.25 |
| overall direct healthcare costs: inpatient care, 1 year after insulin initiation^1^ | | 3712.72 |
| overall direct healthcare costs: ambulatory care, 1 year after insulin initiation^1^ | | 8749.88 |
| overall direct healthcare costs: transport, 1 year after insulin initiation^1^ | | 476.37 |
| overall direct healthcare costs: medical devices, 1 year after insulin initiation^1^ | | 1249.68 |
| overall direct healthcare costs: laboratory tests, 1 year after insulin initiation^1^ | | 343.93 |
| overall direct healthcare costs: paramedical services, 1 year after insulin initiation^1^ | | 2806.97 |
| overall direct healthcare costs: drugs, 1 year after insulin initiation^1^ | | 2586.94 |
| overall direct healthcare costs: physician consultation, 1 year after insulin initiation^1^ | | 1128.98 |

Table 29: Data for France from Jones et al. 2012

| **Jones et al. 2012** | | |
| --- | --- | --- |
| Patient population | Patients with T2D in the 6 months before and after initiation of insulin therapy | |
| Time period | 2006 | |
| Source of costs | Retrospective chart review (baseline information), prospective data collection by physician | |
| Reported cost category | | Annual costs per patient (€) |
| diabetes-specific direct costs: consultation with general practice professionals (visits and phone calls)^2^ | | 456.65 |
| diabetes-specific direct costs: specialist care for glycaemic control^2^ | | 252.64 |
| diabetes-specific direct costs: oral antidiabetic drugs^2^ | | 476.81 |
| diabetes-specific direct costs: insulin^2^ | | 250.27 |
| diabetes-specific direct costs: blood glucose monitoring^2^ | | 315.50 |
| diabetes-associated direct costs: hospitalisation^2^ | | 1366.39 |
| diabetes-specific direct costs: other diabetes related treatment (visits/calls to dieticians, ophthalmologists, chiropodists, podiatrists)^2^ | | 110.31 |
| diabetes-specific direct costs: diabetes treatment (total)^2,*^ | | 3229.75 |

References of tables 5-29

Anderten, H; Dippel, FW; Kostev, K (2015). Early discontinuation and related treatment costs after initiation of Basal insulin in type 2 diabetes patients: a German primary care database analysis. J Diabetes Sci Technol 9(3): 644-50.

Drabik, A; Büscher, G; Sawicki, PT; Thomas, K; Graf, C; Müller, D; Stock, S (2012). Life prolonging of disease management programs in patients with type 2 diabetes is cost-effective. Diabetes Res Clin Pract 95(2): 194-200.

Fritzen, K; Basinska, K; Rubio-Almanza, M; Nicolucci, A; Kennon, B; Vergès, B; Zakrzewska, K; Schnell, O (2019). Pan-European Economic Analysis to Identify Cost Savings for the Health Care Systems as a Result of Integrating Glucose Monitoring Based Telemedical Approaches Into Diabetes Management. J Diabetes Sci Technol 13(3): 1112-1122.

Hessel, F; Freckmann, G; Resch, A (2014). Konsequenzen der Genauigkeit von Blutglucosemesssystemen. Der Diabetologe 10(1): 26-35.

Jacob, L; von Vultee, C; Kostev, K (2017). Prescription Patterns and the Cost of Antihyperglycemic Drugs in Patients With Type 2 Diabetes Mellitus in Germany. J Diabetes Sci Technol 11(1): 123-127.

Jacobs, E; Hoyer, A; Brinks, R; Icks, A; Kuss, O; Rathmann, W (2017). Healthcare costs of Type 2 diabetes in Germany. Diabet Med 34(6): 855-861.

Jones, S; Castell, C; Goday, A; Smith, HT; Nicolay, C; Simpson, A; Salaun-Martin, C (2012). Increase in direct diabetes-related costs and resource use in the 6 months following initiation of insulin in patients with type 2 diabetes in five European countries: data from the INSTIGATE study. Clinicoecon Outcomes Res 4: 383-93.

Kähm, K; Laxy, M; Schneider, U; Rogowski, WH; Lhachimi, SK; Holle, R (2018). Health care costs associated with incident complications in patients with type 2 diabetes in Germany. Diabetes Care 41(5): 971-978.

Liebl, A; Breitscheidel, L; Benter, U; Nicolay, C; Otto, T; Haupt, A (2012). Start der Insulintherapie bei Typ-2-Diabetes: 1-Jahres-Daten der INSTIGATE-Studie. Diabetes, Stoffwechsel und Herz 21(1): 13-9.

Liebl, A; Seitz, L; Palmer, AJ (2014). Health economics analysis of insulin aspart vs. regular human insulin in type 2 diabetes patients, based on observational real life evidence from general practices in Germany. Exp Clin Endocrinol Diabetes 122(9): 517-22.

McDonell, AL; Kiiskinen, U; Zammit, DC; Kotchie, RW; Thuresson, PO; Nicolay, C; Haslam, T; Bruinsma, M; Janszen-Van Oosterhout, AJ; Otto, T (2015). Estimating the real world daily usage and cost for exenatide twice daily and liraglutide in Germany, the Netherlands, and the UK based on volumes dispensed by pharmacies. Clinicoecon Outcomes Res 7: 95-103.

Müller, N; Heller, T; Freitag, MH; Gerste, B; Haupt, CM; Wolf, G; Müller, UA (2015). Healthcare utilization of people with type 2 diabetes in Germany: an analysis based on health insurance data. Diabet Med 32(7): 951-7.

Sittig, DT; Friedel, H; Wasem, J (2015). Prevalence and treatment costs of type 2 diabetes in Germany and the effects of social and demographical differences. Eur J Health Econ 16(3): 305-11.

Ulrich, S; Holle, R; Wacker, M; Stark, R; Icks, A; Thorand, B; Peters, A; Laxy, M (2016). Cost burden of type 2 diabetes in Germany: results from the population-based KORA studies. BMJ Open 6(11): e012527.

Waldeyer, R; Brinks, R; Rathmann, W; Giani, G; Icks, A (2013). Projection of the burden of type 2 diabetes mellitus in Germany: a demographic modelling approach to estimate the direct medical excess costs from 2010 to 2040. Diabet Med 30(8): 999-1008.

Wilke, T; Böttger, B; Berg, B; Groth, A; Botteman, M; Yu, S; Fuchs, A; Maywald, U (2016). Healthcare Burden and Costs Associated with Urinary Tract Infections in Type 2 Diabetes Mellitus Patients: An Analysis Based on a Large Sample of 456,586 German Patients. Nephron 132(3): 215-26.

Baudot, FO; Aguadé, AS; Gastaldi-Ménager, C; Fagot-Campagna, A (2019). Impact of type 2 diabetes on health expenditure: estimation based on individual administrative data. Eur J Health Econ 20(5): 657-668.

Charbonnel, B; Simon, D; Dallongeville, J; Bureau, I; Gourmelen, J; Detournay, B (2018). Direct medical costs of type 2 diabetes in France: An insurance claims database analysis. Medecine des Maladies Metaboliques 11: 11S24-11S27.

Detournay, B; Bureau, I; Gourmelen, J (2015). Le coût de l’insulinothérapie chez les patients diabétiques de type 2, en France. Medecine des Maladies Metaboliques 9: 3S30-3S33.+

Detournay, B (2017). Costs of diabetes drug therapies in France. Medecine des Maladies Metaboliques 11: 11S20-11S23.

Detournay, B; Halimi, S; Torreton, E; Levy, P (2017). Coût des hospitalisations pour hypoglycémie en France chez les patients diabétiques de type 2: étude fondée sur le PMSI et la base EGB de la CNAMTS. Medecine des Maladies Metaboliques 11: 11S16-11S19.

Druet, C; Bourdel-Marchasson, I; Weill, A; Eschwege, E; Penfornis, A; Fosse, S; Fournier, S; Chantry, M; Attali, C; Lecomte, P; Simon, D; Poutignat, N; Gautier, A; Risse, M; Fagot-Campagna, A (2013). Le diabète de type 2 en France : Épidémiologie, évolution de la qualité de la prise en charge, poids social et économique. Entred 2007 Presse Medicale 42(5): 830-838.

Durand-Zaleski, I (2013). Évaluation économique des stratégies et des produits de santé: de la théorie à la pratique Medecine des Maladies Metaboliques 7(3): 220-223.

Hanaire, H; Attali, C; Lecointre, B; Fraysse, M; Gouet, D; Babel, M-R; Charbonnel, B; Sarkozy, F; Gourmelen, J; Detournay, B (2016). Déterminants des coûts du passage à l’insuline en France chez le patient diabétique de type 2: quelles pistes d’optimisation? Sante publique 28(6): 781-789.
